# Unravelling the plasma proteome: Pioneering biomarkers for differential dementia diagnosis

**DOI:** 10.1002/alz.70162

**Published:** 2025-07-04

**Authors:** Haşim Gezegen, Merve Alaylıoğlu, Erdi Şahin, Owen Swann, Elena Veleva, Gamze Güven, Umran Yaman, Derviş A. Salih, Başar Bilgiç, Haşmet Hanağası, Hakan Gürvit, Murat Emre, Duygu Gezen‐Ak, Erdinç Dursun, Henrik Zetterberg, John Hardy, Amanda Heslegrave, Maryam Shoai, Bedia Samanci

**Affiliations:** ^1^ Department of Neurodegenerative Disease UCL Institute of Neurology London UK; ^2^ Behavioral Neurology and Movement Disorders Unit Department of Neurology Istanbul Faculty of Medicine Istanbul University Capa Istanbul Turkey; ^3^ Brain and Neurodegenerative Disorders Research Laboratories Department of Neuroscience Institute of Neurological Sciences Istanbul University‐Cerrahpasa Istanbul Turkey; ^4^ Department of Genetics Aziz Sancar Institute of Experimental Medicine Istanbul University Istanbul Turkey; ^5^ UK Dementia Research Institute at UCL London UK; ^6^ Department of Psychiatry and Neurochemistry Institute of Neuroscience and Physiology the Sahlgrenska Academy at the University of Gothenburg Mölndal Sweden; ^7^ Clinical Neurochemistry Laboratory Sahlgrenska University Hospital Mölndal Sweden; ^8^ Hong Kong Center for Neurodegenerative Diseases Hong Kong China; ^9^ Wisconsin Alzheimer's Disease Research Center University of Wisconsin School of Medicine and Public Health University of Wisconsin‐Madison Madison Wisconsin USA

**Keywords:** AD, DLB, FTD, dementia, plasma biomarkers, pTau

## Abstract

**INTRODUCTION:**

Diagnosing Alzheimer's disease (AD) is challenging due to overlapping symptoms with other dementias and the invasiveness of current biomarkers. This study introduces the NULISA platform, a novel proteomics technology, to evaluate diagnostic accuracy of known biomarkers and uncover novel biomarkers underlying different dementias.

**METHODS:**

We analyzed plasma and cerebrospinal fluid (CSF) samples from 248 participants diagnosed with Alzheimer's disease (AD), dementia with Lewy bodies (DLB), frontotemporal dementia (FTD), and mild cognitive impairment (MCI). Plasma biomarkers were evaluated using regression models, receiver operating characteristics curve (ROC) analysis, and pathway enrichment.

**RESULTS:**

Plasma phosphorylated Tau217 (pTau217) demonstrated the highest diagnostic accuracy for AD, DLB, and FTD (area under the curve [AUCs]: 0.9, 0.84, and 0.79, respectively). CXCL1 (fractalkine), synaptosomal‐associated protein 25 (SNAP25), triggering receptor expressed on myeloid cells 1 (TREM1), β‐synuclein, and tyrosine kinase (TEK) are expressed differently in DLB and FTD than AD. Ingenuity pathway analyses revealed astrocytic, synaptic, and inflammatory pathways as shared and distinct mechanisms across these dementia types.

**CONCLUSION:**

Our findings establish plasma pTau217 as a robust diagnostic marker. This study provides new plasma biomarkers for differential diagnosis of dementias with a noninvasive method.

**Highlights:**

- Plasma pTau217 showed high diagnostic accuracy for AD, DLB, and FTD.
- CXCL1, SNAP25, TREM1, β‐synuclein, and TEK are novel markers distinguishing other dementias from AD.
- Noninvasive plasma biomarkers enable diagnosis and differentiation of dementias.

## 1 BACKGROUND

Alzheimer's disease (AD) is the most common age‐related neurodegenerative dementia, affecting 60%–80% of dementia patients. Its prevalence continues to rise with aging populations, creating an urgent need for accurate diagnosis and effective management. Other dementias, such as dementia with Lewy bodies (DLB) and frontotemporal dementia (FTD), often overlap with AD in symptoms and pathology,^1^ leading to misdiagnosis in up to 30% of cases.^2^ Despite advancements, current diagnostic methods remain challenging, with nearly one‐third of clinically diagnosed AD cases lacking neuropathological confirmation.^2^ This highlights the critical need for reliable and accessible biomarkers.

CSF and neuroimaging biomarkers are essential in AD diagnosis. Cerebrospinal fluid (CSF) markers such as Aβ42 (or Aβ42/40), phosphorylated tau (pTau), and total tau (tTau) reflect key aspects of AD pathology but struggle to fully capture its biological diversity or differentiate it from other dementias with overlapping pathologies.^3^, ^4^ Comprehensive CSF proteome analysis holds promise for identifying novel markers to enhance understanding of AD's complex pathophysiology.^5^ Diagnostic challenges persist for conditions like DLB, where α‐synuclein (SNCA) pathology overlaps with AD in over 40% of cases, complicating differentiation.^6^ Core CSF biomarkers also show limited accuracy in distinguishing DLB from AD due to frequent co‐pathologies, which worsen clinical outcomes and accelerate disease progression.^7^, ^8^ Co‐pathologies with FTD further exacerbate neurodegeneration and complicate diagnosis and treatment.^9^

AD‐related pathophysiological changes can be detected through CSF analysis, positron emission tomography (PET) imaging, and structural magnetic resonance imaging (MRI).^10^ However, their widespread use in primary care is limited by invasiveness (CSF), radiation/cost (PET), and accessibility/cost‐effectiveness, especially in resource‐limited settings where dementia incidence is rising.^11^ This lack of reliable and accessible biomarkers poses a significant challenge, made more urgent by the emergence of disease‐modifying treatments. Plasma biomarkers present a cost‐effective, noninvasive alternative for AD diagnosis, understanding, and management.^11^, ^12^ They enable therapeutic evaluation, risk assessment in asymptomatic individuals, and longitudinal monitoring. Plasma pTau and amyloid biomarkers have shown strong associations with AD pathology and cognitive decline.^13^ Additionally, plasma glial fibrillary acidic protein (GFAP) and neurofilament light chain (NfL) have emerged as potential biomarkers for AD and other neurodegenerative diseases, further expanding the potential of blood‐based diagnostics.^14^ Monoclonal antibody therapies like lecanemab highlight the need for biomarker confirmation, underscoring the shift towards early diagnosis and treatment.^15^ Given the high AD prevalence, increasing demand for therapies, and the need for clinical trials, innovative models for connecting individuals with resources are crucial. Despite challenges, blood‐based biomarkers remain critical for improving diagnosis, therapy, and disease management.

There are a few methods to evaluate plasma biomarkers in dementia. NUcleic acid Linked Immuno‐Sandwich Assay (NULISA), a platform using antibody‐based measurements with a sequencing output, offers advantages such as low sample volume requirements and high multiplexing capacity.^16^ Its central nervous system (CNS) panel includes traditional AD, synaptic, microglial, neuroinflammation, and emerging neurodegeneration biomarkers, demonstrating effective detection and accuracy.^17^

This study utilized NULISA to analyze 120 plasma biomarkers across diverse biological mechanisms in a well‐characterized cohort, including patients with mild cognitive impairment (MCI), AD, DLB, and FTD from a single referral memory center. The primary objectives were: (1) to assess this innovative technology, validate existing diagnostic biomarkers, and identify novel ones to enhance diagnostic precision; and (2) to explore plasma proteomic changes underlying the pathogenesis of AD, DLB, and FTD, identifying new biomarker candidates for future investigation.

## 2 METHODS

### 2.1 Patients

Patients were recruited from Behavioral Neurology and Movement Disorders Unit, Department of Neurology, Istanbul Faculty of Medicine, Istanbul University, a referral center for cognitive disorders. The study was conducted in accordance with the ethical standards of the Declaration of Helsinki and approved by the Istanbul University Ethics Committee (approval number 2023/1191, granted 22/06/2023). We included Caucasian patients born in Turkiye.

The study encompassed a comprehensive assessment of participants, including medical history, physical and neurological examinations, psychiatric screening, clinical laboratory tests, and cranial magnetic resonance imaging. The neuropsychological evaluation included the Mini‐Mental State Examination and the Clinical Dementia Rating Scale. A subset of patients also completed a comprehensive neuropsychological assessment at baseline.

Experienced clinicians made diagnoses based on established criteria,^10^, ^18^ which included progressive cognitive complaints corroborated by an informant, clinically observed cognitive impairment as previously described, clinical laboratory tests and MRI findings that excluded other potential causes of impairment as well as CSF draw. Genetic causes were excluded for the FTD group with a gene panel that consists of known FTD genes and C9Orf72 repeat expansion.

**RESEARCH IN CONTEXT**

1. **Systematic review**: The authors reviewed the literature on plasma and cerebrospinal fluid (CSF) biomarkers for diagnosing and differentiating dementia types, using sources such as PubMed and recent conference proceedings. Emerging evidence highlights plasma pTau217, glial fibrillary acidic protein (GFAP), and neurofilament light chain (NfL) as promising noninvasive diagnostic tools for Alzheimer's disease (AD) and other dementias. Additionally, the role of novel proteomic alterations in dementia pathophysiology remains an area of active research, supported by relevant citations.
2. **Interpretation**: Our findings confirm plasma pTau217 as a robust biomarker for diagnosing AD, dementia with Lewy bodies (DLB), and frontotemporal dementia (FTD) with high accuracy. Novel plasma biomarkers, including CXCL1 (fractalkine), synaptosomal‐associated protein 25 (SNAP25), triggering receptor expressed on myeloid cells 1 (TREM1), β‐synuclein, and tyrosine kinase (TEK), were differentially expressed across dementia types, supporting their potential role in differential diagnoses. These results align with known pathways, such as integrin signaling and synaptic modulation, implicated in neurodegeneration.
3. **Future directions**: The study emphasizes the need for validation of these findings in larger, diverse cohorts. Future research should explore (a) the longitudinal dynamics of plasma biomarkers in early and progressive dementia; (b) the mechanistic role of inflammatory and synaptic pathways in dementia progression; and (c) the integration of plasma biomarkers with other noninvasive diagnostic modalities to improve early detection and personalized treatment strategies.

Inclusion criteria for the study were age 55 years or older and signed informed consent for biological sample extraction and storage. Exclusion criteria included major systemic or psychiatric illness, significant sensory deprivation, and contraindications for the required complementary tests. Patients who underwent CSF examination between June 23, 2023, and March 25, 2024, and met the inclusion criteria were included in the study.

### 2.2 CSF biomarkers and NULISA measurements

All participants underwent a lumbar puncture (LP) to measure Aβ42, pTau181, tTau, and NfL levels, as well as blood sample collection.

Following an overnight fast, LP was performed at 09.00 am using a 25‐G needle. Ten milliliter of CSF, collected in four polypropylene tubes, was kept on ice for up to 1 h before centrifugation at 2000 g for 10 min at 4°C. Previously mentioned biomarkers were run on the same day. Aliquots (250 µL) were stored in 1‐mL polypropylene tubes at ‐80°C until analysis.

Blood samples, collected concurrently with LP, were processed within 1 h. Plasma was obtained by collecting 10 mL of blood into ethylenediaminetetraacetic acid (EDTA) tubes, inverting five times, and centrifuging at 2000 g for 10 min at 4°C. Aliquots (250 µL) were stored in 1‐mL polypropylene tubes at ‐80°C.

The CSF levels of Aβ42, pTau181, tTau, and NfL were measured at Brain and Neurodegenerative Disorders Laboratory, Department of Neuroscience, Institute of Neurological Sciences, Istanbul University‐Cerrahpasa, which is an active member of The Alzheimer's Association Quality Control (AAQC) program. The measurements were done with INNOTEST β‐AMYLOID (1‐42) ELISA kit (81576, FUJIREBIO), INNOTEST PHOSPHO‐TAU (181P) ELISA Kit (81574, FUJIREBIO), INNOTEST hTAU Ag ELISA Kit (81572, FUJIREBIO), and NF‐light ELISA kit (10‐7001, UmanDiagnostics), respectively. All measurements were performed according to the manufacturer's assay protocol, and each sample and standard were tested in duplicate.

Plasma biomarkers were measured using the NULISA platform, developed by Alamar Biosciences, following the protocol established in ref. 16

### 2.3 Statistical analysis

All analyses were performed in R 4.2.2. Data were log2‐transformed and tested for normality (NPQ values from NULISA were already log_2_ transformed and no additional transformation was used). One‐way analysis of variance (ANOVA) or Welch's ANOVA was used to compare normally distributed data, followed by calculation of effect sizes (η^2^p). The Kruskal–Wallis test was used for non‐normally distributed data. A‐T‐N‐ MCI patients who are accepted as ‘biological healthy controls’ further analyzed for biomarker levels to highlight differences relative to AD, DLB, and FTD.

To evaluate diagnostic accuracy of plasma pTau217 and other biomarkers, the dataset was divided into training and testing sets. A repeated 10‐fold cross‐validation was employed. All models adjusted for age and sex. Predictive performance comparisons for biomarkers were performed by using receiver operating characteristic (ROC) curves and calculating the area under the curve (AUC). Differences between AUC values were tested with the DeLong test. Cutoff values maximizing Youden indices were used for sensitivity and specificity. The analysis was conducted with *caret* and *pROC* packages.

Spearman correlations with false discovery rate (FDR) correction were employed to conduct head‐to‐head comparisons between CSF and plasma biomarkers, as well as between plasma biomarkers themselves. Scatter plots with linear regression lines investigated the relationship between CSF and plasma biomarkers.

We applied principal component analysis (PCA) to the plasma biomarkers to assess the contributions of individual proteins and to explore whether the most discriminative features could clearly separate diagnostic groups. We then extracted the variable loadings for the first two principal components (PCs) and visualized these with bar plots. We used the *prcomp* function for PCA analysis.

Multinomial logistic regression assessed the association between plasma biomarkers and disease categories. A separate model was fitted for each biomarker. Odds ratios (ORs) were calculated to quantify the likelihood of a specific diagnosis relative to the biomarker levels. Adjusted *p*‐values were computed using the FDR method. Also, A‐T‐N‐ MCI patients are taken as controls and compared with disease groups. We performed an age and sex adjusted ordinal regression model to assess the associations between plasma biomarkers and disease stage in the AD continuum. These models were conducted using *nnet* and *MASS* packages.

Random forest analyses were conducted separately with all biomarkers and biomarkers that differed between diagnosis based on multinomial logistic regression and calculated the importance of all features to evaluate the differential diagnostic performance of the discriminating proteins identified through regression analyses and to propose a clinically applicable protein panel. All features were tested with 10‐fold cross‐validation that a balanced distribution of age and gender across the subsets and mean AUC was used as the performance metric. The model was trained with the method argument set to “*rf*”. The analysis was conducted using *caret* package.

Ingenuity pathway analysis (IPA) was used to investigate molecular mechanisms distinguishing DLB and FTD from AD. Protein‐level expression data were used from multinomial regression model to estimate associations with DLB and FTD, using AD as the reference group. Proteins with significant associations (adjusted *p*‐value < 0.05, FDR correction applied) were selected for IPA. Regulatory networks specific to DLB and FTD were created, alongside a combined DLB/FTD network, to visualize shared and distinct molecular mechanisms relative to AD. IPA predicted key upstream regulators, downstream targets, and signaling pathways, focusing on activated or inhibited pathways in DLB and FTD while emphasizing overlaps and distinctions from AD.

## 3 RESULTS

### 3.1 Clinical and demographical features

We analyzed data from 248 subjects (130 females, 118 males) with a mean age of 66.7 (± 9.1) years and an mini‐mental state examination (MMSE) of 21.7 (± 7.1). Diagnoses included 117 AD, 50 MCI, 39 FTD, 25 DLB, and 17 other dementias. Other dementias consisted of NPH (normal pressure hydrocephalus), CBS (corticobasal syndrome), and PSP (progressive supranuclear palsy). Table 1 details MMSE, Clinical Dementia Rating scale (CDR), and CSF amyloid (A), tau (T), and neurodegeneration (N) group distribution by diagnosis, showing no sex differences. The DLB and FTD groups differ from other groups for age and the MCI group differed from other groups for MMSE. Table 2 presents demographics for AD continuum patients.

**TABLE 1:** Demographics of the cohort.

Variable	Overall *N* = 248	AD *N* = 117	MCI *N* = 50	FTD *N* = 39	DLB *N* = 25	Other *N* = 17	*p*‐value^a^
Age, mean (SD)	66.7 (9.1)	66.5 (8.5)	66.2 (9.6)	61.9 (8.6)	74.7 (5.6)	68.9 (8.9)	<0.001
Female *n* (%)	118 (48%)	53 (45%)	25 (50%)	16 (41%)	16 (64%)	8 (47%)	0.4
MMSE, mean (SD)	21.7 (7.1)	20.4 (7.1)	27.5 (2.0)	19.8 (7.9)	20.3 (6.5)	19.3 (8.9)	<0.001
CDR, range (median)	0–3 (0.5)	0–3 (0.5)	0–0.5 (0.5)	0–3 (1)	0.5–2 (0.5)	0.5–2 (1)	
ATN, *n* (%)							
A‐T‐N‐	46 (19%)	0 (0%)	20 (40%)	12 (31%)	9 (36%)	5 (29%)	
A‐T‐N+	5 (2.0%)	0 (0%)	2 (4.0%)	3 (7.7%)	0 (0%)	0 (0%)	
A‐T+N‐	11 (4.4%)	0 (0%)	8 (16%)	1 (2.6%)	1 (4.0%)	1 (5.9%)	
A‐T+N+	27 (11%)	0 (0%)	12 (24%)	10 (26%)	2 (8.0%)	1 (5.9%)	
A+T‐N‐	40 (16%)	5 (4.3%)	5 (10%)	11 (28%)	10 (40%)	9 (53%)	
A+T‐N+	2 (0.8%)	1 (0.9%)	0 (0%)	0 (0%)	1 (4.0%)	0 (0%)	
A+T+N‐	7 (2.8%)	5 (4.3%)	2 (4.0%)	0 (0%)	0 (0%)	0 (0%)	
A+T+N+	110 (44%)	106 (91%)	1 (2.0%)	2 (5.1%)	2 (8.0%)	1 (5.9%)	

*Note*: A: CSF amyloid β42, T: CSF phosphorylated Tau181, N: CSF total tau. Others include NPH, CBS, and PSP.

Abbreviations: AD, Alzheimer dementia; ANOVA, analysis of variance; CBS, corticobasal syndrome; CDR, Clinical Dementia Rating scale; DLB, dementia with Lewy bodies; FTD, frontotemporal dementia; MCI, mild cognitive impairment; MMSE, Mini‐Mental State Examination; NPH, normal pressure hydrocephalus; PSP, progressive supranuclear palsy; SD, standard deviation.

^a^ ANOVA/Welch's ANOVA; Pearson's chi‐squared test.

**TABLE 2:** Demographics of Alzheimer's disease stages.

Variable	Overall *N* = 102	Stage 2 *N* = 8	Stage 3 *N* = 15	Stage 4 *N* = 34	Stage 5 *N* = 31	Stage 6 *N* = 14	*p*‐value^a^
Age, mean (SD)	66.1 (9.0)	67.6 (13.1)	69.7 (6.0)	66.7 (8.4)	65.4 (9.7)	61.5 (8.0)	0.12
Sex, female (%)	53 (52%)	2 (25%)	9 (60%)	17 (50%)	19 (61%)	6 (43%)	0.4
MMSE, mean (SD)	21.0 (7.1)	28.3 (2.1)	28.2 (1.3)	24.6 (2.8)	18.0 (1.9)	8.2 (3.8)	<0.001
CDR, range (median)	0–3 (0.5)	0	0.5	0–1(0.5)	0.5–2(1)	1–3 (2)	
ATN, n (%)							
A+T‐N‐	9 (8.8%)	5 (63%)	4 (27%)	0 (0%)	0 (0%)	0 (0%)	
A+T‐N+	1 (1.0%)	0 (0%)	0 (0%)	0 (0%)	1 (3.2%)	0 (0%)	
A+T+N‐	6 (5.9%)	2 (25%)	2 (13%)	1 (2.9%)	0 (0%)	1 (7.1%)	
A+T+N+	86 (84%)	1 (13%)	9 (60%)	33 (97%)	30 (97%)	13 (93%)	

*Note*: A: CSF amyloid β42, T: CSF phosphorylated Tau181, N: CSF total tau.

Abbreviations: CDR, Clinical Dementia Rating scale; MMSE, Mini‐Mental State Examination; SD, standard deviation.

^a^ Kruskal–Wallis rank sum test; Fisher's exact test.

Twenty A‐T‐N‐ MCI patients had a mean age of 63.5 (± 10.7) years, and half of them were female. The mean MMSE score was 28.2 (± 1.5), and the median CDR was 0 (0–0.5). The mean CSF Aβ42 level was 1045.6 pg/ml (± 120.1), CSF pTau181 was 36.6 pg/ml (± 4.7), tTau was 215.4 pg/ml (± 53.9), and NfL was 591.8 pg/ml (± 214.5).

### 3.2 Diagnostic and differential diagnosis performance of plasma biomarkers

The mean NPQ (NULISA Protein Quantification) values of pTau217 showed significant differences between AD and the other groups, with a very large effect size (*p* < 0.001; η^2^p (partial eta squared) = 0.45). DLB patients had 1.3 times higher pTau217 NPQ values than MCI patients (*p* = 0.03). The NPQ values of pTau181 and pTau231 between AD patients and other groups was also significantly different (*p* < 0.001; η^2^p = 0.30 with a 95% confidence interval (CI) (0.22–1) and *p* < 0.001; η^2^p = 0.37 with a 95% CI (0.29–1), respectively). Like pTau217, pTau181, and pTau231 were significantly higher in DLB patients compared to MCI (*p* = 0.003 and *p* = 0.001, respectively). FTD patients’ mean NPQ values of pTau231 were also slightly higher than MCI patients (*p* = 0.04).

Plasma mean Aβ42 NPQ values was highest in the DLB group with a moderate effect size (*p* = 0.002; η^2^p = 0.07 with a 95% confidence interval [CI] (0.02–1), *p*‐values for DLB vs. AD was 0.009, DLB vs. MCI 0.008, DLB vs. FTD 0.002).

The median NfL NPQ value was highest in FTD patients with relatively large effect size (*p* < 0.001; ε2 = 0.2 with a 95% CI (0.12–1)). AD and DLB group's median NfL levels were higher than the MCI group's (1.5 times difference with *p* < 0.001‐ and 5‐times difference with *p* < 0.001, respectively).

**FIGURE 1:**
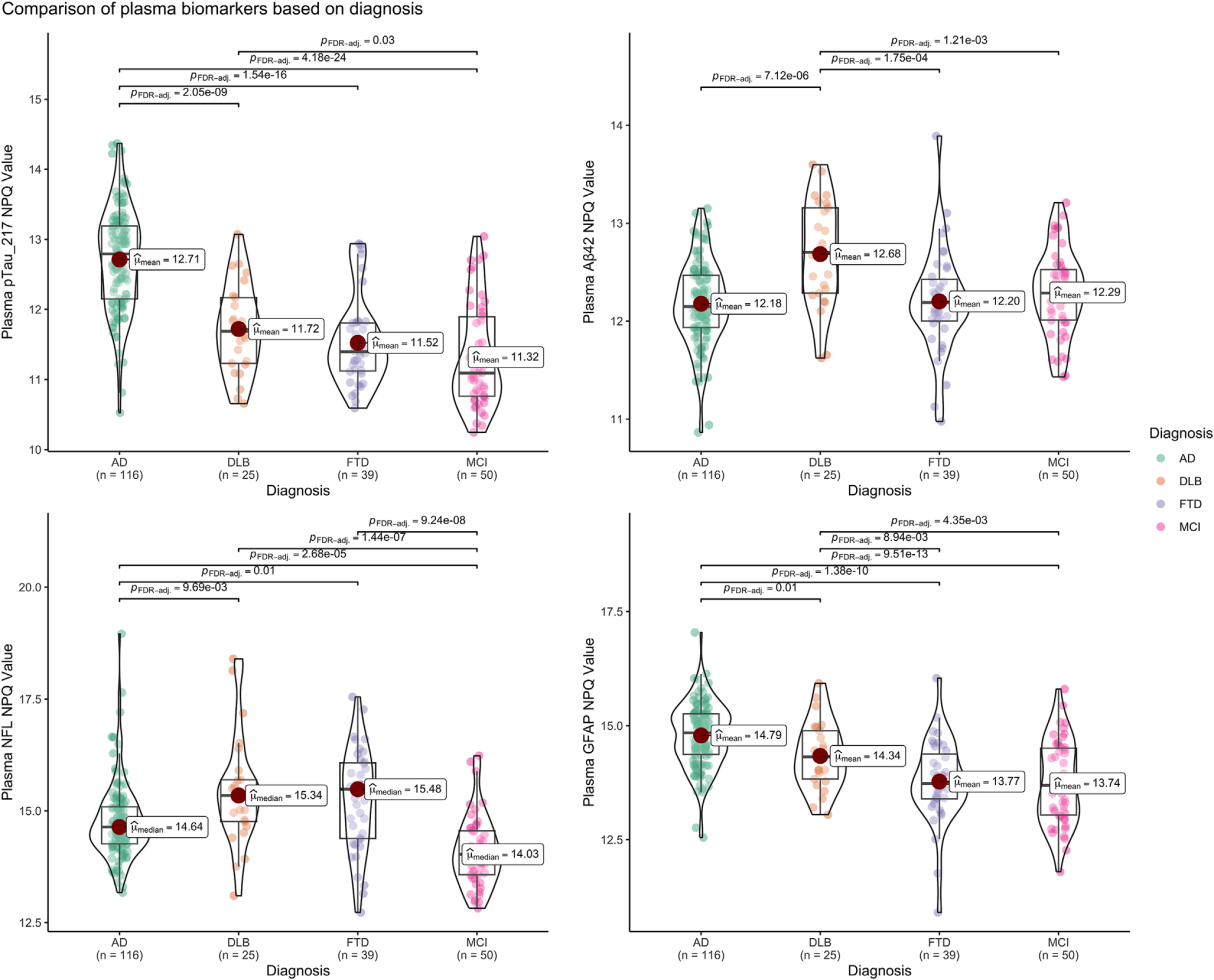
Violin plots illustrating plasma biomarker levels of pTau217, Aβ42, NfL, and GFAP across patients with AD, DLB, FTD, and MCI. Each violin plot shows the distribution of normalized NPQ values for each biomarker within the diagnostic groups, with mean or median values highlighted by red squares. Statistically significant differences between diagnostic groups were assessed using adjusted *p*‐values (*p* < 0.05, FDR‐adj indicated) from group comparisons. Aβ42, amyloid‐beta 42; AD, Alzheimer's disease; DLB, dementia with Lewy bodies; FDR‐adj, post hoc pairwise Dunn's test with false discovery rate adjustment; FTD, frontotemporal dementia; GFAP, glial fibrillary acidic protein; MCI, mild cognitive impairment; NfL, neurofilament light chain; NPQ, normalized protein quantification; pTau‐217, phosphorylated tau‐217.

The mean plasma GFAP NPQ value was highest in the AD group with a large effect size (*p* < 0.001; η^2^p = 0.28 with a 95% CI (0.19–1). Also, it was higher in the DLB group compared to the MCI and FTD groups (1.5 times difference with *p* = 0.004‐ and 1.5‐times difference with *p* = 0.009, respectively) (Figure 1).

A‐T‐N‐ MCI patients who are classified as controls compared with AD, DLB, and FTD patients for pTau217, Aβ42, NfL, and GFAP and further enhanced the differentiation except for Aβ42 (Supplementary Material 1)

**FIGURE 2:**
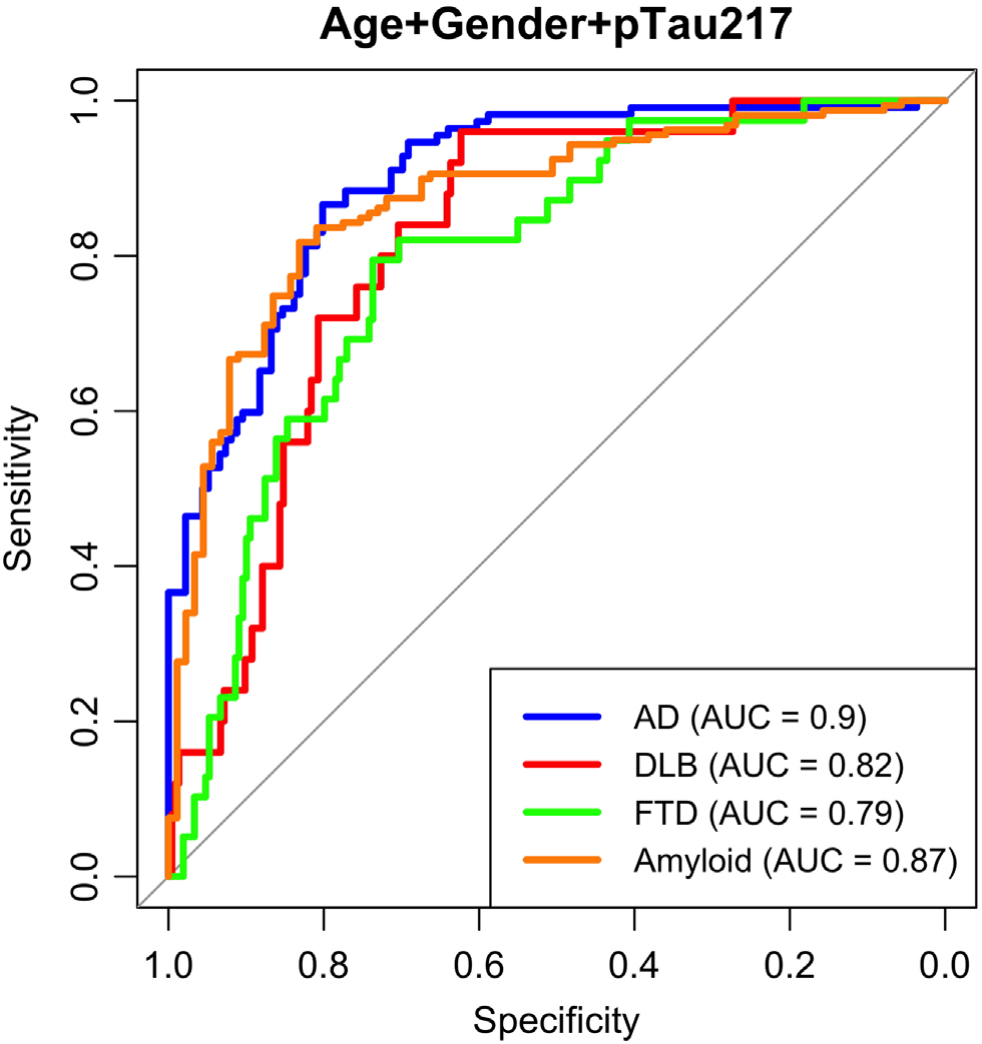
ROC curves for CSF amyloid status, AD, DLB, and FTD diagnosis. The ROC curves display the diagnostic performance of a model combining plasma pTau217 levels, age, and sex for predicting AD, DLB, FTD diagnoses, and amyloid status in CSF, separately. The AUC for AD, DLB, FTD, and amyloid positivity are 0.9, 0.82, 0.79, and 0.87, respectively. AD, Alzheimer's disease; AUC, area under the curve; CSF, cerebrospinal fluid; DLB, dementia with Lewy bodies; FTD, frontotemporal dementia; ROC, receiver operating characteristic.

We evaluated the ability of different plasma markers to detect amyloid pathological change (A+) defined by CSF and diagnosis. When assessing the potential of plasma pTau217 values to differentiate between A+ and A− status, AUC was 0.87 (95%CI 0.83–0.92) with 0.82 sensitivity and 0.83 specificity. Accuracy was 0.82 PPV (positive predictive value) 0.89 and negative predictive value (NPV) 0.72 (Figure 2). An NPQ value of 11.9 for pTau217 was used as the cutoff value to differentiate between A+ and A‐ status.

Plasma pTau217 value showed the best performance for predicting AD (AUC 0.9 [95%CI 0.86–0.94], 0.87 sensitivity and 0.8 specificity with accuracy 0.79, PPV 0.81, and NPV 0.78. An NPQ value of 11.9 for pTau217 was used as the cutoff value to differentiate AD vs. other groups), DLB (AUC 0.84 [95%CI 0.76–0.91], 0.8 sensitivity and 0.8 specificity with accuracy 0.9, PPV 0.5, and NPV 0.9, an NPQ value of 12.7 for pTau217 was used as the cutoff value to differentiate DLB vs. other groups) and FTD diagnosis (AUC 0.79 [95%CI 0.72–0.86], 0.79 sensitivity and 0.74 specificity with accuracy 0.83, PPV 0 and NPV 0.84, an NPQ value of 11.8 for pTau217 was used as the cutoff value to differentiate FTD vs. other groups). ORs of these models are shown in (Supplementary Material 2).

Next, we studied how the combination of different markers predicted amyloid status. Adding GFAP to pTau217 increased the AUC to 0.89 (95%CI 0.84–0.93) but there were no differences between models (*p* = 0.12). Also adding Aβ40, Aβ42, or Aβ42/40 data did not improve the model. FABP3 (fatty acid‐binding protein) had a similar performance as pTau217 for DLB diagnosis (AUC 0.83 [95%CI 0.76–0.90], 0.84 sensitivity and 0.75 specificity). When FABP3 was added pTau217 the AUC was 0.87 (95%CI 0.8–0.94) but this did not improve the model (*p* = 0.08). GFAP in DLB also showed a similar performance for diagnosis (AUC 0.82 [95%CI 0.75–0.89]).

When adding NfL data to pTau217 for FTD diagnosis the AUC increased to 0.88 (95% CI 0.83–0.92) with 0.97 sensitivity and 0.67 specificity *p* < 0.01).

### 3.3 Plasma and CSF biomarker correlation

**FIGURE 3:**
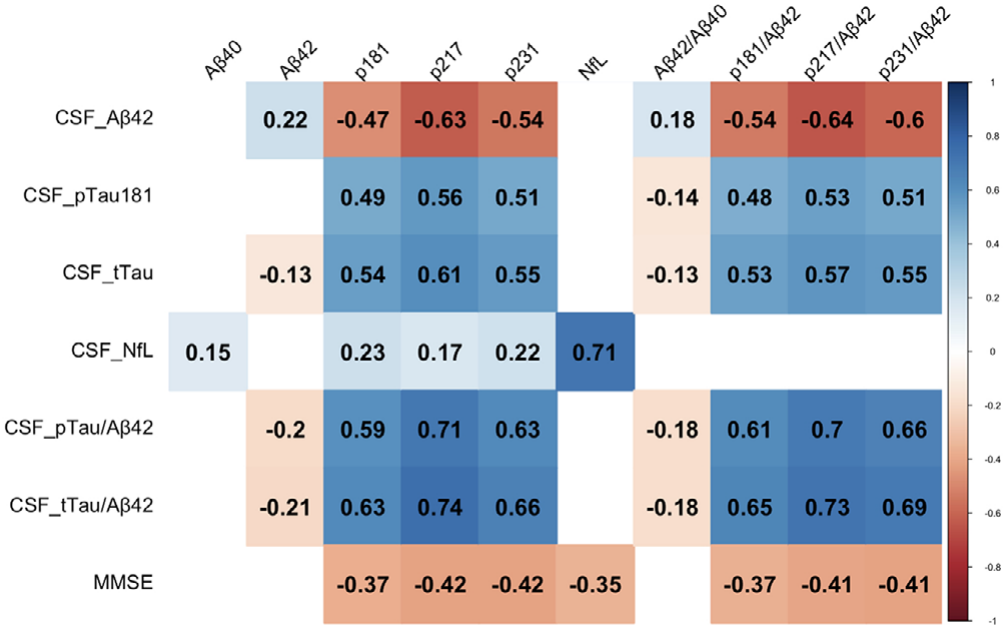
Correlation heatmap between CSF and plasma biomarkers for all cohort. The heatmap displays the correlation between CSF biomarkers and MMSE on the *y*‐axis and plasma biomarkers on the *x*‐axis. The numbers represent Spearman correlation coefficients, indicating the strength and direction of the relationships between the variables. Blue indicates positive correlations, and red indicates negative correlations. Only FDR corrected *p*‐value < 0.05 are showed. Aβ42, amyloid‐beta 42; Aβ40, plasma amyloid‐beta 40; CSF, cerebrospinal fluid; FDR, false discovery rate; MMSE, Mini‐Mental State Examination; NfL, neurofilament light chain; p181, phosphorylated tau‐181; p217, phosphorylated tau‐217; p231, phosphorylated tau‐231; pTau181, phosphorylated tau 181; pTau/Aβ42, ratio of phosphorylated tau 181 to Aβ42; Aβ42/Aβ40, ratio of Aβ42 to Aβ40; p181/Aβ42, ratio of phosphorylated tau 181 to Aβ42; p217/Aβ42, ratio of phosphorylated tau 217 to Aβ42; p231/Aβ42, ratio of phosphorylated tau 231 to Aβ42; tTau, total tau; tTau/Aβ42, ratio of total tau to Aβ42.

In the overall cohort, CSF Aβ42 negatively correlated with plasma pTau, with a slight improvement for plasma pTau/Aβ42. CSF pTau181 and tTau positively correlated with plasma pTau, with a correction for CSF Aβ42 enhancing these relationships. MMSE moderately correlated with plasma pTau and NfL (Figure 3).

When analyzed by diagnosis, CSF Aβ42 positively correlated with plasma Aβ42 in DLB and FTD, and with plasma Aβ40 only in DLB. In AD, CSF Aβ42 negatively correlated with plasma pTau217. Correcting plasma pTau for Aβ42 strengthened the correlations in FTD and DLB. The strongest CSF‐plasma pTau correlations were observed in DLB, with a further improvement after Aβ42 correction. Notably, A+TxN‐ (CSF amyloid+, Tau+ or ‐, and neurodegeneration‐) MCI showed markedly increased CSF pTau181 and plasma pTau correlations. CSF pTau181 correlated with the Aβ42/Aβ40 ratio in DLB. CSF tTau correlated with plasma pTau in AD and DLB, with the strongest correlations in A+TxN‐ MCI for plasma pTau181. CSF NfL consistently correlated with plasma NfL across all groups (Supplementary Material 3).

All plasma pTau biomarkers correlated with CSF pTau181/Aβ42 in DLB and AD, with stronger correlations in DLB. While correlations for CSF and plasma biomarkers were low in AD patients, their trajectories through the AD continuum displayed a similar pattern (Supplementary Material 4).

Regarding CSF AT status, CSF Aβ42 moderately correlated with plasma Aβ42/Aβ40 only in A‐T+. It negatively correlated with plasma pTau biomarkers in A+T‐ and A‐T+ groups, with no correlations in A‐T‐ or A+T+. Correcting plasma pTau for Aβ42 improved A+T‐ and A‐T+ correlations and revealed correlations in A+T+ group (Supplementary Material 5).

CSF Aβ42 negatively correlated with GFAP in DLB. CSF NFL correlated with plasma GFAP in AD and negatively correlated with plasma GFAP/NfL in AD, DLB, and FTD. CSF NfL also correlated with plasma tTau and FABP3 in DLB, and with FABP3 in FTD. CSF tTau correlated with plasma total Tau and FABP3 in DLB, and with tTau and GFAP in A+TxN‐ MCI. MMSE was negatively correlated with all plasma pTau with the strongest correlation observed for pTau217 (*r* = ‐0.51, *p* < 0.01). Additionally, MMSE negatively correlated with plasma GFAP, tTau, and NfL in this group. In the DLB group MMSE is negatively correlated with plasma GFAP and tTau, and MMSE is negatively correlated with GFAP in the FTD group.

Plasma huntingtin (HTT), SNCA, superoxide dismutase‐1 (SOD1), and TARDBP biomarkers were highly correlated with each other (HTT vs. SNCA *r* = 0.89, HTT vs. SOD1 *r* = 0.87, HTT vs. TARDBP *r*:0.93, SNCA vs. SOD1 *r*: 0.9, SNCA vs. TARDBP *r*:0.87, SOD1 vs. TARDPB *r*: 0.83; all adjusted *p*‐values < 0.001) (Supplementary Material 6). And all of them were highly correlated with fibroblast growth factor 2 (FGF2), ARSA, ANXA5, interleukin (IL) 18, MDH1, PGK1, RUVBL2 (*r*:0.64–0.92). These high correlations persisted, or even increased, when subdividing different disease groups.

### 3.4 Biomarker differentiation across neurodegenerative diseases and AD stages

**FIGURE 4:**
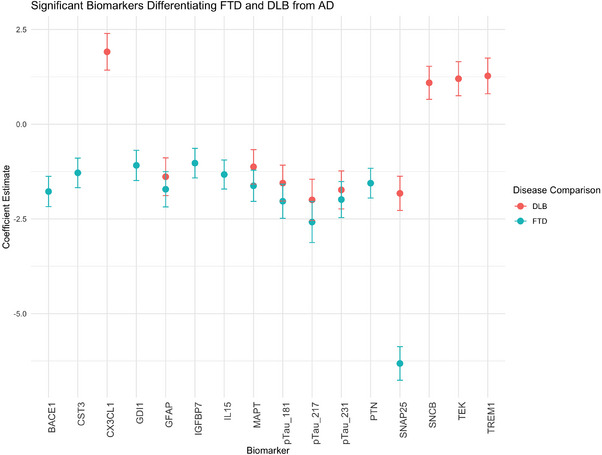
Multinomial logistic regression coefficients for biomarkers differentiating DLB and FTD from AD. Biomarkers that above zero are increased in DLB compared to AD. Biomarkers that below zero are increased in AD compared to DLB or FTD. AD, Alzheimer's disease; BACE1, beta‐site amyloid precursor protein cleaving enzyme 1; CST3, cystatin C; CX3CL1, chemokine (C‐X3‐C motif) ligand 1 (fractalkine); DLB, dementia with Lewy bodies; FTD, frontotemporal dementia; GDI1, GDP dissociation inhibitor 1; GFAP, glial fibrillary acidic protein; IGFBP7, insulin‐like growth factor binding protein 7; IL15, interleukin‐15; MAPT, microtubule‐associated protein tau; pTau181, phosphorylated Tau‐181; pTau217, phosphorylated Tau‐217; pTau231, phosphorylated Tau‐231; PTN, pleiotrophin; SNAP25, synaptosomal‐associated protein 25; SNCB, beta synuclein; TEK, tyrosine kinase; TREM1, triggering receptor expressed on myeloid cells 1.

In comparison with the AD group, plasma pTau181, pTau217, and pTau231 had significantly lower odds for DLB (OR 0.21, 95% CI: 0.08–0.53, *p* = 0.005; OR 0.14, 95% CI: 0.05–0.4, *p* = 0.001; and OR 0.18, 95% CI: 0.07–0.47, *p* = 0.003, respectively) and for FTD group (OR 0.13, 95% CI: 0.05–0.32, *p* < 0.001; OR 0.08, 95% CI: 0.05–0.35, *p* < 0.001; and OR 0.14, 95% CI: 0.05–0.35, *p* < 0.001, respectively), controlling for age and sex (Figure 4). Total tau and GFAP also showed lower odds for both DLB (OR 0.32 and 0.25) and FTD (OR 0.2 and 0.18) groups.

In comparison with the AD group, β‐synuclein (SNCB), tyrosine kinase (TEK), and triggering receptor expressed on myeloid cells 1 (TREM1) had higher ORs for the DLB group. When compared with AD; beta‐secretase‐1 (BACE1), cystatin C (CST3), GDP‐dissociation inhibitor‐1 (GDI1), insulin‐like GF‐binding protein‐7 (IGFBP7), IL15, pleiotrophin (PTN), and synaptosomal‐associated protein 25 (SNAP25) had lower odds for the FTD group. The DLB group also had lower odds for SNAP25.

When we compare the disease groups with A‐T‐N‐ MCI group we found pTau181, pTau217, pTau231, GFAP, and NPTX2 were increased only in AD, SNCB was increased only in DLB. NfL and TEK were increased in all three disease groups when compared to A‐T‐N‐ MCI, GDI1, and tTau increased both AD and DLB, CXCL1 (fractalkine) was increased both DLB and FTD. CST3 and SNAP25 were decreased in FTD when comparing A‐T‐N‐ MCI (Supplementary Material 7)

**FIGURE 5:**
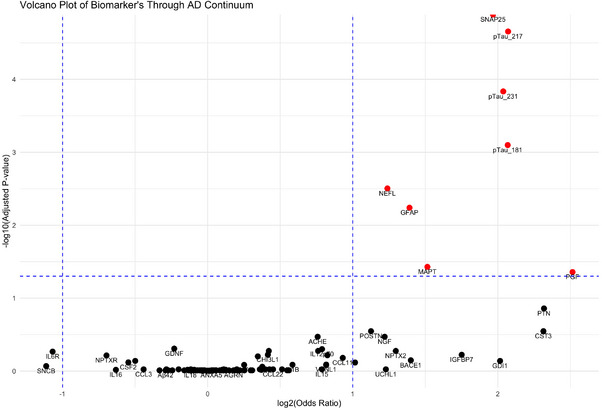
Volcano plot of biomarkers across the AD continuum based on ordinal regression. Red biomarkers are significantly increasing with disease stage. AD, Alzheimer's disease; GFAP, glial fibrillary acidic protein; MAPT, microtubule‐associated protein tau; NEFL, neurofilament light chain; PGF, placental growth factor; pTau181, phosphorylated tau‐181; pTau217, phosphorylated tau‐217; pTau231, phosphorylated tau‐231; SNAP25, synaptosomal‐associated protein 25.

In the analysis of biomarkers across the AD continuum, placental growth factor (PGF) was significantly associated with disease stage, with an OR of 5.7 (95% CI:1.8–18, *p* = 0.002), indicating a 5.7‐fold increase in odds for more advanced stages per unit increase in PGF levels. Similarly, pTau217 (OR = 4.2, CI: 2.4–7.3, *p* < 0.001), pTau181 (OR = 4.2, CI:2.2–8.2, *p* < 0.001), pTau231 (OR = 4.1, CI: 2.3–7.5, *p* < 0.001), SNAP25 (OR = 3.9, CI:3.2–4.8, *p* < 0.001), tTau (OR = 2.9, CI:1.5–5.6, *p* = 0.002), GFAP (OR = 2.6, CI: 1.6–4.4, *p* < 0.001), and NfL (OR = 2.4, CI: 1.5–3.7, *p* < 0.001) showed a significant association with disease progression (Figure 5).

We extracted all plasma biomarkers with age and sex into the random forest classification model and calculated feature importance. ROC analysis for the diagnosis of AD, FTD, and DLB and the top 20 features are shown in Supplementary Material 8. The accuracy of this model was 0.73.

ROC‐AUC of all features for distinguishing AD from other groups was 0.85 [95% CI: 0.81–0.88], FTD from other groups was 0.83 [95% CI: 0.79–0.88] and DLB from other groups was 0.75 [95% CI: 0.69–0.82] (Supplementary Material 9). The first 10 important biomarkers for AD, FTD, and DLB are shown in Supplementary Material 10–12.

**FIGURE 6:**
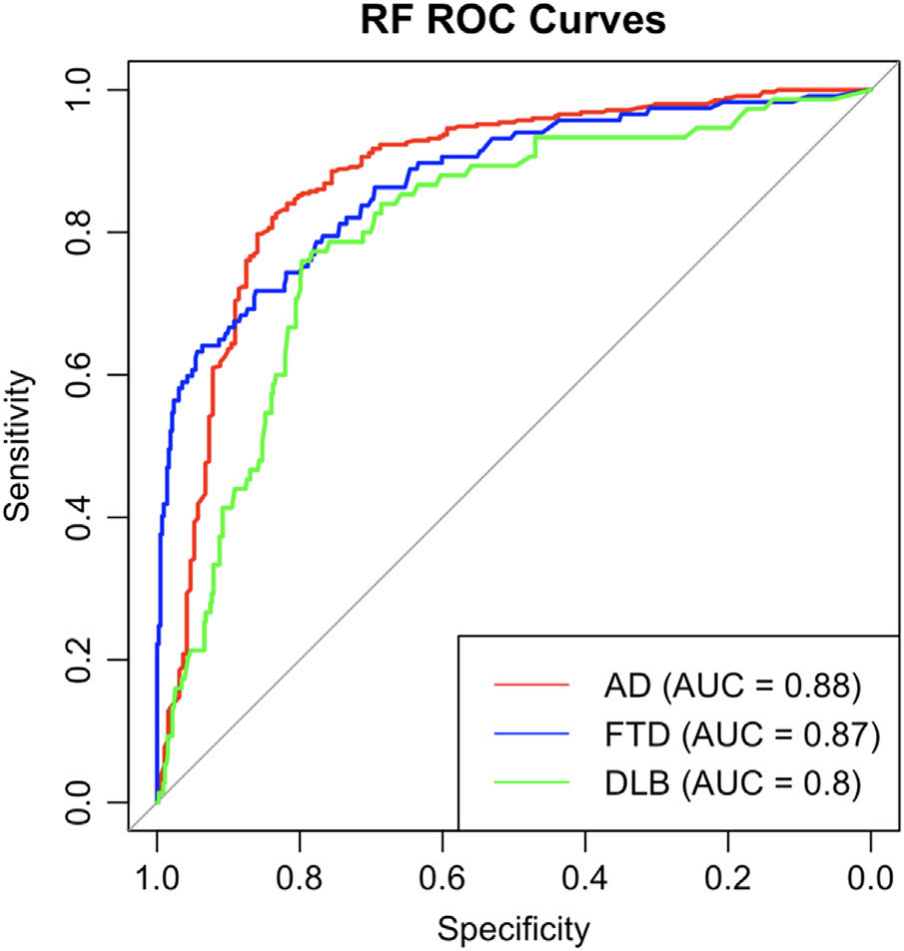
RF ROC curve for dementia diagnosis, differentiated biomarkers. The ROC curves display the diagnostic performance of a regression model combining only differently expressed plasma biomarkers, age, and sex for predicting AD, DLB, FTD diagnoses. The AUC for AD, FTD, and DLB are 0.88, 0.87, and 0.8, respectively. AD, Alzheimer's disease; AUC, area under the curve; DLB, dementia with Lewy bodies; FTD, frontotemporal dementia; RF, random forest; ROC, receiver operating characteristic.

When we took the biomarkers that we find significant in the multinomial logistic regression model, our random forest model improved for all categories. AUC for AD was 0.88 [95% CI: 0.84–0.91], FTD was 0.87 [95% CI: 0.83–0.91], and DLB was 0.81 [95% CI: 0.76–0.86] (Figure 6). Accuracy for the whole model was 0.76. According to this model, the biomarkers are ranked in order of importance and are provided in Supplementary Material 13–15.

### 3.5 PCA

The PCA revealed that the first two PCs (PC1 and PC2) accounted for nearly 30% of the total variance (e.g., PC1 explained 18.4% and PC2 10.9%). The biplot demonstrated little separation between DLB and other groups (Supplementary Material 16). The loadings for responsible PC1 and PC2 plotted in Supplementary Material 17.

### 3.6 IPA

The DLB‐specific network highlighted significant roles for SNAP25, tTau, and GFAP as central regulators with significant roles in synaptic and inflammatory pathways, and decreased in DLB compared to AD. The SNCA pathway was unique to DLB. Predicted activation of the phosphoinositide 3‐kinase/protein kinase B (PI3K/AKT) and N‐methyl‐D‐aspartate (NMDA) receptor signaling suggests links between synaptic dysfunction and neuroinflammation. Increased TREM1 and cytokine signaling pathways reflect the inflammatory burden in DLB, while mitogen‐activated protein kinase (MAPK)/extracellular signal‐regulated kinase 1/2 (ERK1/2), and AKT serine/threonine kinase 1 (AKT1) activation points to cellular survival mechanisms compensating for synaptic loss (Supplementary Material 18).

FTD‐specific network revealed enrichment of tau‐related pathways, with tTau as a network driving role, the inhibition of these pathways suggests the relative activation in AD. While SNAP25 and GFAP remained significant, the absence of the SNCA pathway distinguishes FTD from DLB. The FTD network (Supplementary Material 19) displayed fewer inflammatory pathways but more focused interactions involving ERK1/2 signaling.

In the combined network (Supplementary Material 20), shared features of DLB and FTD were integrated while maintaining distinctions. Shared pathways, including ERK1/2 and AKT1 signaling, were active in both conditions.

## 4 DISCUSSION

In this study, we investigated plasma biomarkers associated with neurodegeneration, to assess their diagnostic accuracy and correlation with established CSF biomarkers and conducted exploratory analyses within a mixed dementia cohort. Plasma pTau217 emerged as a key biomarker across AD, DLB, and FTD. Additionally, plasma GFAP and NfL correlated with disease severity and distinguished between diagnostic groups. Novel plasma proteomic alterations were identified for different dementia types, providing promising biomarker candidates for future research.

Consistent with previous studies, plasma pTau217, pTau181, pTau231, as well as GFAP, exhibited the highest NPQ values in the AD group, while Aβ42 was highest in DLB group.^19^ Across the entire cohort, these plasma pTau biomarkers strongly correlated with CSF Aβ42, pTau181, and tTau, with correlations further enhanced when pTau values were adjusted for Aβ42 in both plasma and CSF. Additionally, CSF and Plasma NfL demonstrated robust correlations.

Plasma pTau217 demonstrated the strongest diagnostic performance across AD, DLB, and FTD, with an AUC of 0.9, 0.84, and 0.79, respectively. It also accurately predicted CSF amyloid positivity (AUC = 0.87). These findings align with a recent study employing 33 different pTau biomarker assays, discriminative accuracies for detecting AD pathology, and inter‐platform agreement for plasma pTau217 measurements compared to other plasma pTau variants.^20^ Plasma pTau217, evaluated on the NULISA platform, achieved an AUC of 0.91 for amyloid‐PET positivity.^21^ These studies collectively highlight plasma pTau biomarkers, particularly pTau217, as the most sensitive plasma biomarkers for AD diagnosis, even in its early stages.^22^

The association between AD progression and pTau217, pTau181, pTau231, tTau, NfL, and GFAP aligns with the literature.^23^, ^24^, ^25^, ^26^ Our findings support studies indicating SNAP25^27^ and PGF, a VEGF family member, in disease progression.^28^ Adding NfL to pTau217 improved FTD diagnostic accuracy (AUC = 0.88), while plasma GFAP and FABP3 were comparable to pTau217 in differentiating DLB. These findings are consistent with previous reports ^25^, ^29^ and highlight the utility of combining multiple biomarkers to enhance diagnostic accuracy.

Plasma pTau and NfL negatively correlated with MMSE scores in the AD group, reflecting their association with cognitive decline.^23^, ^24^ Plasma GFAP levels also showed negative correlations with MMSE across AD, DLB, and FTD, indicating its role as a marker of disease severity.^30^, ^31^ In the DLB group, plasma GFAP and pTau levels were elevated compared to MCI, and CSF Aβ42 negatively correlated with plasma GFAP. All three plasma pTau forms correlated with CSF pTau181, and pTau217 effectively discriminated against the DLB group. These results align with studies evaluating amyloid and tau‐PET imaging alongside plasma biomarkers in the DLB continuum.^29^ Unlike the AD group, our analysis revealed a correlation between CSF Aβ42 and plasma Aβ40 and Aβ42 in the DLB group. Although both AD and DLB have amyloid plaques, their composition differs, with DLB plaques containing minimal Aβ40 and showing a broader reduction in amyloid‐beta species.^31^ These findings support studies showing that plasma GFAP can distinguish DLB from other dementias and is associated with amyloid pathology.

Even though we do not have a healthy control group, A‐T‐N‐ MCI individuals, though not strictly “healthy,” provide a biologically relevant comparison group that approximates a cognitively normal population. This group showed similar cognitive and CSF profiles to healthy controls in literature.^18^ Studies have demonstrated that A‐T‐N‐ MCI individuals do not show the hallmark pathological alterations of AD and tend to have biomarker levels that closely resemble those of A‐T‐N‐ cognitively normal individuals.^3^, ^12^ We further analyzed biomarker levels in A‐T‐N‐ MCI to highlight differences relative to AD, DLB, and FTD. Levels of pTau217, NfL, and GFAP were more pronounced when comparing the disease groups to the overall MCI group.

When stratified by diagnosis, correlations between plasma pTau and CSF biomarkers diminished in AD, with no significant correlation with CSF pTau. This aligns with prior research showing weak correlations between plasma and CSF pTau, and a dissociation between tau‐PET and CSF pTau in AD.^26^, ^32^ A recent study found no correlations between plasma and CSF pTau levels within AD group.^20^ These findings suggest fluid and imaging biomarkers capture distinct aspects of tau pathophysiology, reflected in the updated ATN classification (T1: CSF/plasma, T2: tau PET/oligomeric Tau).^10^ Imaging, animal, and *post mortem* studies suggest soluble pTau is a better reflection of Aβ42 plaque concentration than PET‐measured or PHF‐tau.^31^, ^33^, ^34^, ^35^ In our study, CSF pTau correlated with plasma pTau only in A+T‐ individuals, consistent with studies showing significance in A+ MCI and cognitively normal A+ individuals.^36^, ^37^ Our findings, consistent with others, demonstrate the strongest CSF/plasma pTau correlation in amyloid‐positive MCI ^11^, ^38^ and a weakening of this correlation in later AD stages. This suggests in early AD stages CSF and plasma pTau are compatible. Also this correlation showed in cognitively normal A+ individuals.^37^ This aligns with the weakening pTau‐NFT correlation in AD dementia and late Braak stages.^36^ We showed CSF and plasma biomarkers in AD exhibit similar trajectories, with pTau and NfL levels initially increasing with disease severity, then plateauing and declining. This reflects early CSF pTau increase and later decrease in symptomatic AD.^36^ Soluble pTau peaks during maximal amyloid load and declines, likely due to sequestration within neurofibrillary tangles (NFTs), mirroring animal models where CSF pTau tracks Aβ deposition.^34^ We also found a correlation between CSF pTau/Aβ42 ratio and plasma pTau. The pTau/Aβ42 ratio improved correlations with DLB, FTD, and amyloid‐positive MCI, supporting its role in AD discrimination and amyloid‐PET prediction.^39^ These findings suggest pTau levels accompany amyloid pathology, with the pTau/Aβ42 ratio offering improved correlation and diagnostic potential.

In this study, while we found high correlations for HTT, SNCA, SOD1, TARDBP (TAR‐DNA‐binding protein‐43), and other biomarkers, these are not typically correlated with CSF and have nonbrain sources.^40^, ^41^ Further investigation is needed to determine whether these correlations are due to peripheral origins, shared enzymatic alterations, or truly informative significant associations.

Our study highlighted distinct biomarker profiles for different dementia types. Plasma CX3CL1, SNCB, TEK, and TREM1 are elevated in DLB compared to AD. CX3CL1, primarily expressed by neurons, plays a role in modulating microglial activity, mediating neuron‐microglia communication, and Parkinson's disease (PD) ‐related inflammation.^42^ However, its role in DLB remains underexplored. Elevated CSF SNCB levels were found only in AD, but not in those with PD or DLB. The absence of SNCB expression in blood cells highlights its potential as a peripheral blood biomarker.^43^ Further investigation is needed due to the limited research in this area. TEK inhibitors, which reduce CSF SNCA, are under investigation for DLB treatment.^44^ CSF TREM1 levels were elevated in AD compared to MCI, with no significant differences between AD and DLB,^45^ highlighting the need for more studies on plasma TREM1 in these conditions. We also found elevated levels of BACE1, CST3 (cystatin‐C), GDI1, GFAP, IGFBP7, IL15, tTau, PGF, pTau, PTN, and SNAP25 (synaptosomal‐associated protein‐25) in AD compared to FTD. GFAP, tTau, and all three pTau biomarkers were also increased in AD compared to DLB, with no differences observed between DLB and FTD patients for these biomarkers. These results align with previously reported in the literature. Others are biomarkers associated with amyloid pathology and AD.^46^, ^47^ These findings suggest that distinct biological processes are dysregulated across different dementias, providing new avenues for therapeutic targeting.

The synaptic and inflammatory pathway inhibition indicates these pathways are more prominently activated in AD relative to DLB could indicate a compensatory mechanism in response to the accumulation of amyloid‐β and tau. The synuclein pathway, PI3K/AKT signaling, and inflammatory responses are central to DLB, while tauopathy and neuronal structural changes define FTD.^48^ Also compared to DLB, in AD, AKT1, and ERK1/2 are inhibited, potentially contributing to greater synaptic dysfunction and reduced cellular resilience.^49^

Random forest analysis reveals that overall importance is primarily driven by pTau and amyloid‐related biomarkers. When evaluating synaptic dysfunction biomarkers in CSF, it was demonstrated that SNAP25, 14‐3‐3 zeta/delta, and NPTX2 (neuronal pentraxin), in various combinations, can discriminate AD from other neurodegenerative diseases.^50^, ^51^ IL33 appears to be linked to tau pathology and microglial activation^51^ and is closely associated with FTD.^52^, ^53^ CHI3L1 (chitinase‐3‐like protein‐1) is increased in the brain of sporadic FTD, while UCHL1 is increased in CSF, and NPTX2 is decreased in CSF both sporadic and genetic FTD.^54^ RF analysis of differently expressed biomarkers also demonstrated good diagnostic accuracy, with AUCs of 0.88, 0.87, and 0.80 for AD, DLB, and FTD, respectively. Combining multiple biomarkers in classification models improved diagnostic accuracy, underscoring the potential of biomarker panels for clinical application.

Even though our predictive models achieved high accuracy, the PCA analysis did not show a clear separation between diagnostic groups. We worked with log_2_‐transformed NPQ values, which were not calibrated against external standards of known absolute concentration. Since PCA is highly dependent on variable scaling, applying dimensionality reduction to this dataset may not have resulted in a clear separation.

Our study has some limitations. The absence of healthy controls limits distinguishing normal aging from pathology. Single‐center recruitment impacts generalizability, and external validation is needed. Co‐pathologies in DLB and FTD may have influenced biomarker profiles, and our younger AD cohort restricts understanding of plasma pTau performance in older populations. However, the study utilized the innovative NULISA platform to analyze 120 plasma biomarkers linked to neurodegeneration, neuroinflammation, and synaptic function. A well‐characterized cohort and advanced statistical methods strengthened the findings, providing insights into biomarker specificity and potential for noninvasive, cost‐effective diagnostics.

This study demonstrates the potential of plasma biomarkers, especially pTau217, GFAP, and NfL, as noninvasive tools for dementia diagnosis and differentiation. Using the NULISA platform and comprehensive statistical methods, the results emphasize their diagnostic accuracy and potential to reveal proteomic changes in dementia. These advances address diagnostic challenges and support early intervention and personalized treatment. Further validation in larger, diverse groups is needed for clinical application and improved differential diagnoses.

## CONFLICT OF INTEREST STATEMENT

H.Z. has served at scientific advisory boards and/or as a consultant for Abbvie, Acumen, Alector, Alzinova, ALZpath, Amylyx, Annexon, Apellis, Artery Therapeutics, AZTherapies, Cognito Therapeutics, CogRx, Denali, Eisai, LabCorp, Merry Life, Nervgen, Novo Nordisk, Optoceutics, Passage Bio, Pinteon Therapeutics, Prothena, Quanterix, Red Abbey Labs, reMYND, Roche, Samumed, Siemens Healthineers, Triplet Therapeutics, and Wave, has given lectures sponsored by Alzecure, BioArctic, Biogen, Cellectricon, Fujirebio, Lilly, Novo Nordisk, Roche, and WebMD, and is a co‐founder of Brain Biomarker Solutions in Gothenburg AB (BBS), which is a part of the GU Ventures Incubator Program (outside submitted work). A.J.H. has consulted Quanterix and Lilly. Author disclosures are available in the Supporting Information.

## CONSENT STATEMENT

All human participants provided informed consent.

## Supporting information



Supporting Information

Supporting Information
