# Supplementary material for: Unravelling the plasma proteome: Pioneering biomarkers for differential dementia diagnosis
Source: Alzheimers Dement. 2025 Jul 4;21(7):e70162. doi: 10.1002/alz.70162 (PMC12231214; doi:10.1002/alz.70162)
Supplement: Supplementary file 1 — Supporting Information [file ALZ-21-e70162-s001.docx]

***
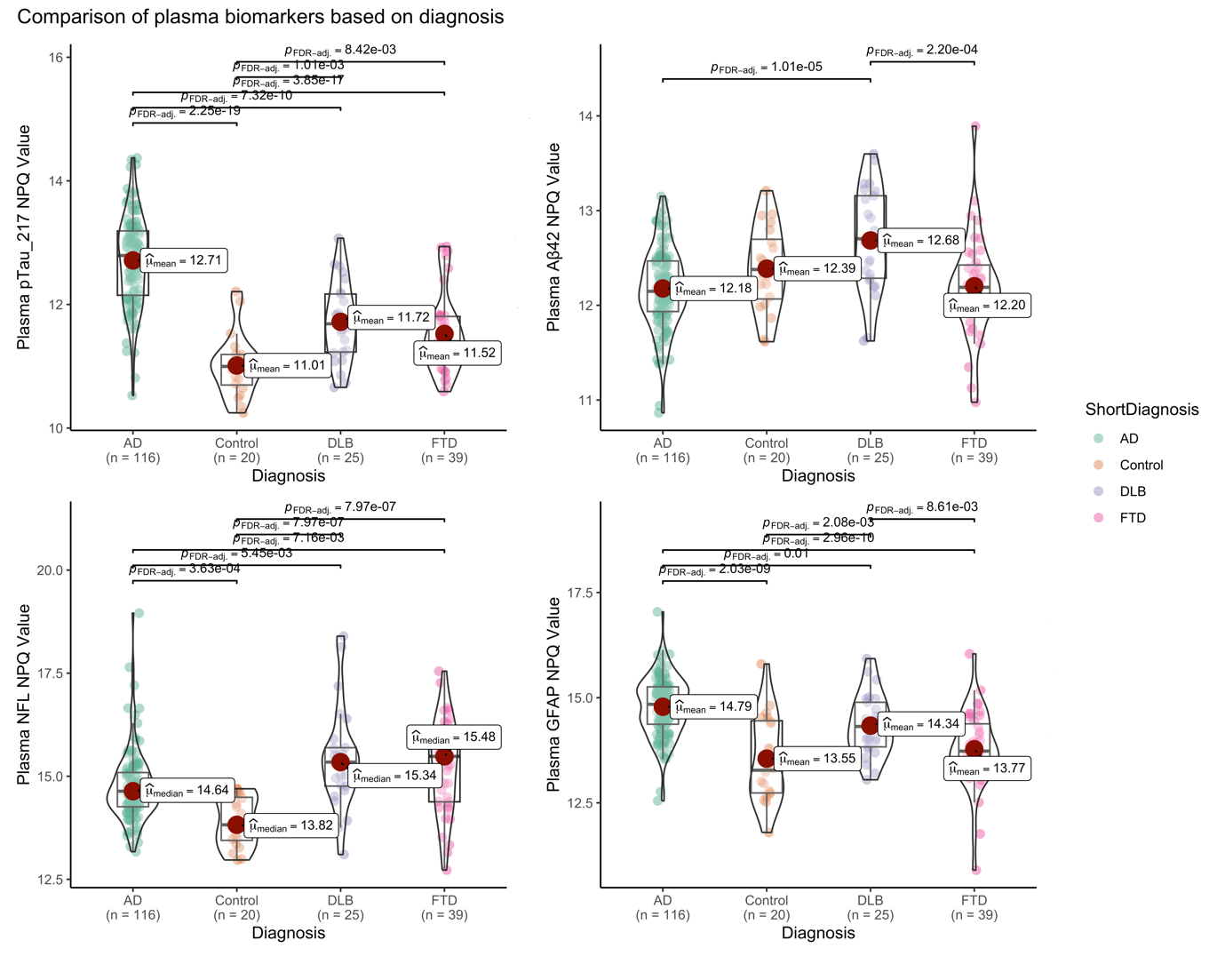
***

***Supp material 1.*** Violin plots illustrating plasma biomarker levels of pTau217, Aβ42, NfL, and GFAP across patients with AD, DLB, FTD, and A-T-N- MCI (classified as ‘Control’). Each violin plot shows the distribution of normalized NPQ values for each biomarker within the diagnostic groups, with mean or median values highlighted by red squares. Statistically significant differences between diagnostic groups were assessed using adjusted p-values (p<0.05, FDR-adj indicated) from group comparisons. NPQ: Normalized Protein Quantification, pTau-217: phosphorylated tau-217, Aβ42: amyloid-beta 42, NfL: neurofilament light chain, GFAP: glial fibrillary acidic protein, AD: Alzheimer’s Disease, DLB: Dementia with Lewy Bodies, FTD: Frontotemporal Dementia, MCI: Mild Cognitive Impairment, FDR-adj: Post hoc pairwise Dunn’s test with FDR (False Discovery Rate) adjustment

|  | CSF Amyloid positivity | | | AD Diagnosis | | | FTD Diagnosis | | | | DLB Diagnosis | | | |
| --- | --- | --- | --- | --- | --- | --- | --- | --- | --- | --- | --- | --- | --- | --- |
| **Characteristic** | **OR**^1^ | **95% CI**^1^ | **p-value** | **OR**^1^ | **95% CI**^1^ | **p-value** | **OR**^1^ | **95% CI**^1^ | **p-value** | **OR**^1^ | | **95% CI**^1^ | **p-value** |  |
| Age | 1.03 | 0.99, 1.07 | 0.2 | 0.99 | 0.95, 1.03 | 0.6 | 1.18 | 1.10, 1.28 | *<0.001* | 0.93 | | 0.89, 0.97 | *<0.001* |  |
| Sex | 0.78 | 0.40, 1.53 | 0.5 | 1.11 | 0.55, 2.27 | 0.8 | 1.72 | 0.69, 4.48 | 0.2 | 0.65 | | 0.30, 1.37 | 0.3 |  |
| pTau_217 | 7.87 | 4.86, 13.7 | *<0.001* | 11.0 | 6.49, 20.1 | *<0.001* | 0.48 | 0.25, 0.85 | *0.011* | 0.42 | | 0.26, 0.63 | *<0.001* |  |
| ***Supp material 2.*** *ROC Models* AD: Alzheimer dementia, MCI: Mild cognitive impairment, FTD: Frontotemporal dementia, DLB: Dementia with Lewy bodies ^1^OR = Odds Ratio, CI = Confidence Interval | | | | | | | | | | | | | | |


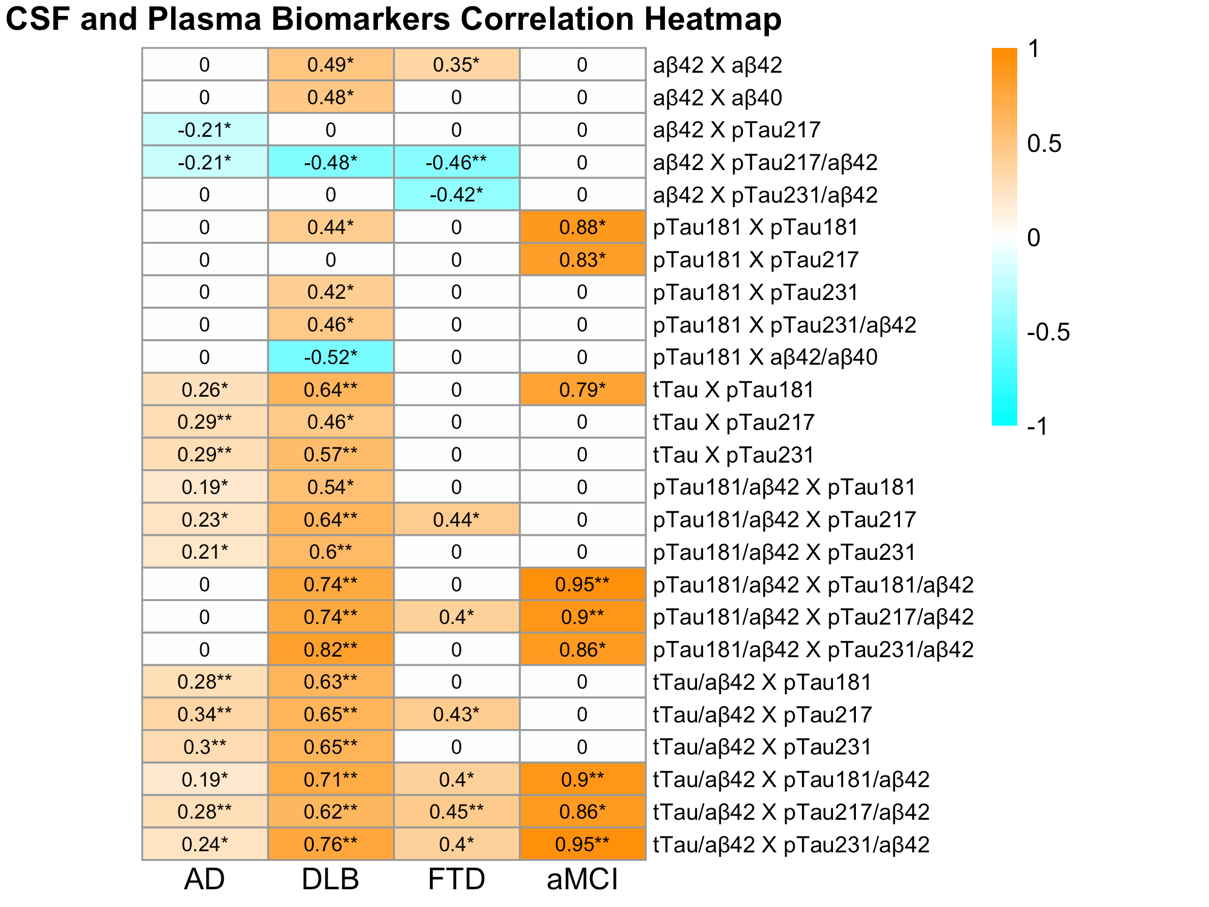
***Supp material 3.*** CSF and plasma biomarkers correlation heatmap, based on diagnosis. The heatmap represents the correlation between CSF and plasma biomarkers across diagnostic groups. First biomarker represents CSF and second biomarker represents plasma. The colour scale represents correlation coefficients, with orange indicating positive correlations and blue indicating negative correlations. AD: Alzheimer’s Disease, DLB: Dementia with Lewy Bodies, FTD: Frontotemporal Dementia, aMCI: amyloid positive Mild Cognitive Impairment, Aβ42: amyloid beta 42, Aβ40: amyloid beta 40, pTau181: phosphorylated Tau-181, pTau217: phosphorylated tau-217, pTau231: phosphorylated tau-231, tTau: total tau. * p<0.05, ** p<0.01


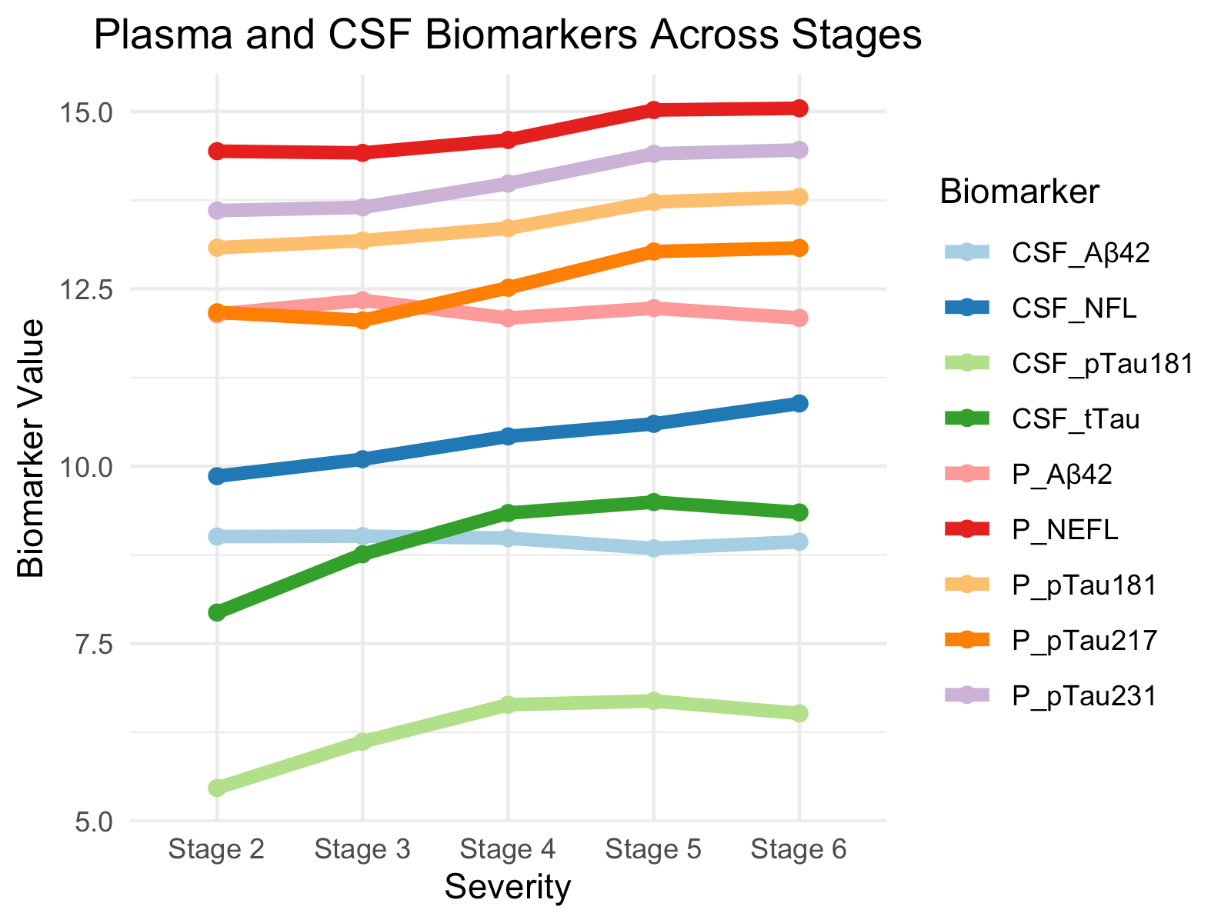


***Supp material 4.*** Plasma and CSF biomarkers’ trajectory across AD continuum. This figure displays the changes in various plasma and CSF biomarkers across AD stages of disease severity. The lines represent the mean biomarker values as the disease progresses, allowing for comparisons between plasma and CSF biomarkers at different stages. CSF_Aβ42: cerebrospinal fluid amyloid beta 42, CSF_NFL: cerebrospinal fluid neurofilament light chain, CSF_pTau181: cerebrospinal fluid phosphorylated tau-181, CSF tTau: cerebrospinal fluid total tau, P_Aβ42: plasma amyloid beta-42, P_NEFL: plasma neurofilament light chain, P_pTau181: plasma phosphorylated tau-181, P_pTau217: plasma phosphorylated tau-217, P_pTau231: plasma phosphorylated tau-231


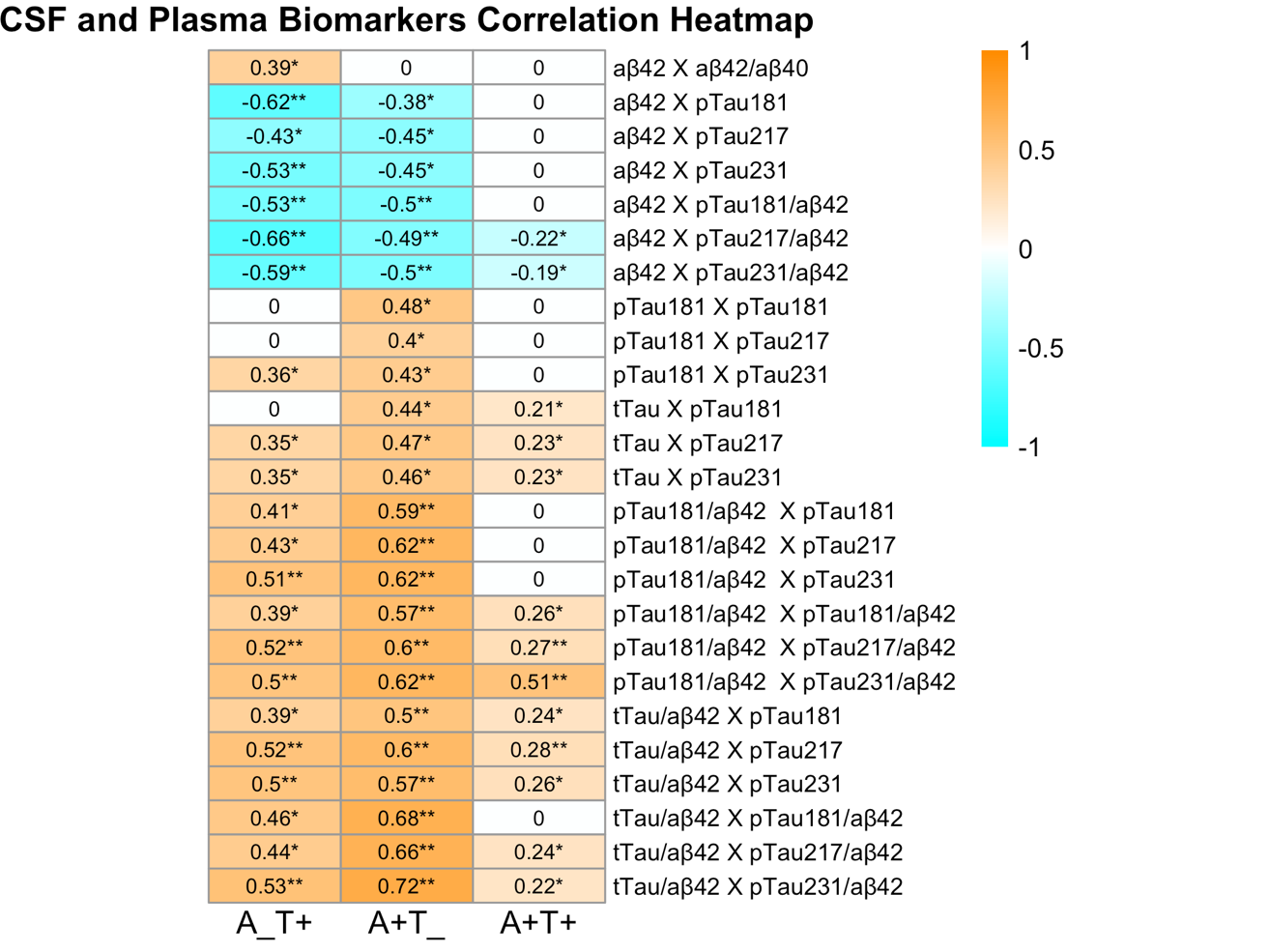


***Supp material 5.*** CSF and plasma biomarkers correlation heatmap, based on CSF AT (amyloid-Tau) status***.*** The heatmap represents the correlation between CSF and plasma biomarkers for different CSF AT status. First biomarker represents CSF and second biomarker represents plasma. The colour scale represents correlation coefficients, with orange indicating positive correlations and blue indicating negative correlations. Aβ42: amyloid beta 42, Aβ40: amyloid beta 40, pTau181: phosphorylated tau-181, pTau217: phosphorylated tau-217, pTau231: phosphorylated tau-231, tTau: total tau. * p<0.05, ** p<0.01

***
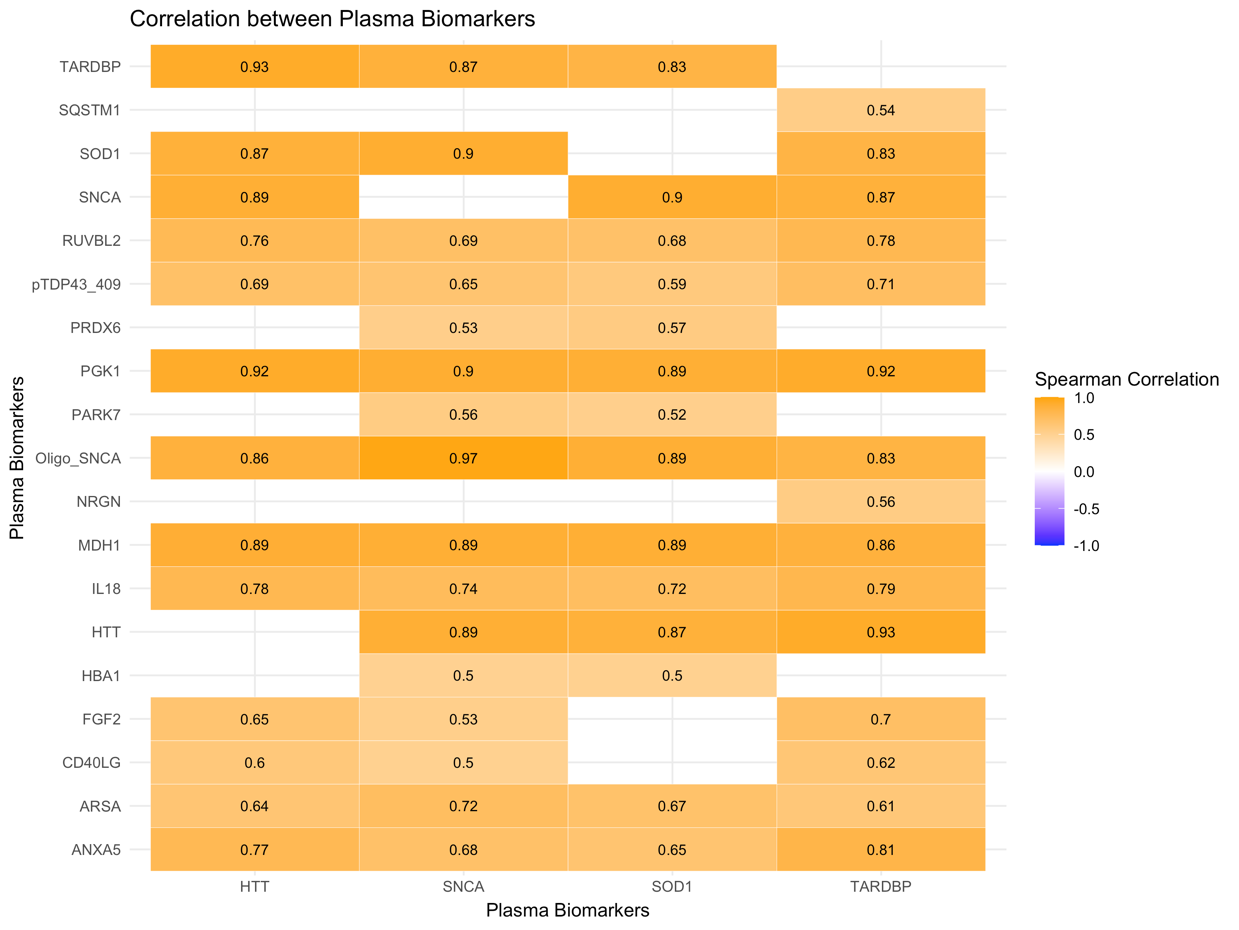
***

***Supp material 6.*** Plasma biomarkers correlation for HTT, SNCA, SOD1 and TARDBP in whole cohort. This heatmap illustrates the Spearman correlation between various plasma biomarkers, highlighting pairwise relationships among markers involved in neurodegeneration. Darker colors indicate higher positive correlations, while lighter shades indicate weaker or no correlation. TARDBP: TAR DNA-binding protein 43, SQSTM1: sequestosome 1, SOD1: superoxide dismutase 1, SNCA: alpha-synuclein, RUVBL2: RuvB-like AAA ATPase 2, pTDP43_409: phosphorylated TAR DNA-binding protein 43 at residue 409, PRDX6: peroxiredoxin 6, PGK1: phosphoglycerate kinase 1, PARK7: parkinsonism associated deglycase (DJ-1), Oligo_SNCA: oligomeric alpha-synuclein, NRGN: neurogranin, MDH1: malate dehydrogenase 1, IL18: interleukin 18, HTT: huntingtin, HBA1: haemoglobin subunit alpha 1, FGF2: fibroblast growth factor 2, CD40LG: CD40 ligand, ARSA: arylsulfatase A, ANXA5: annexin A5


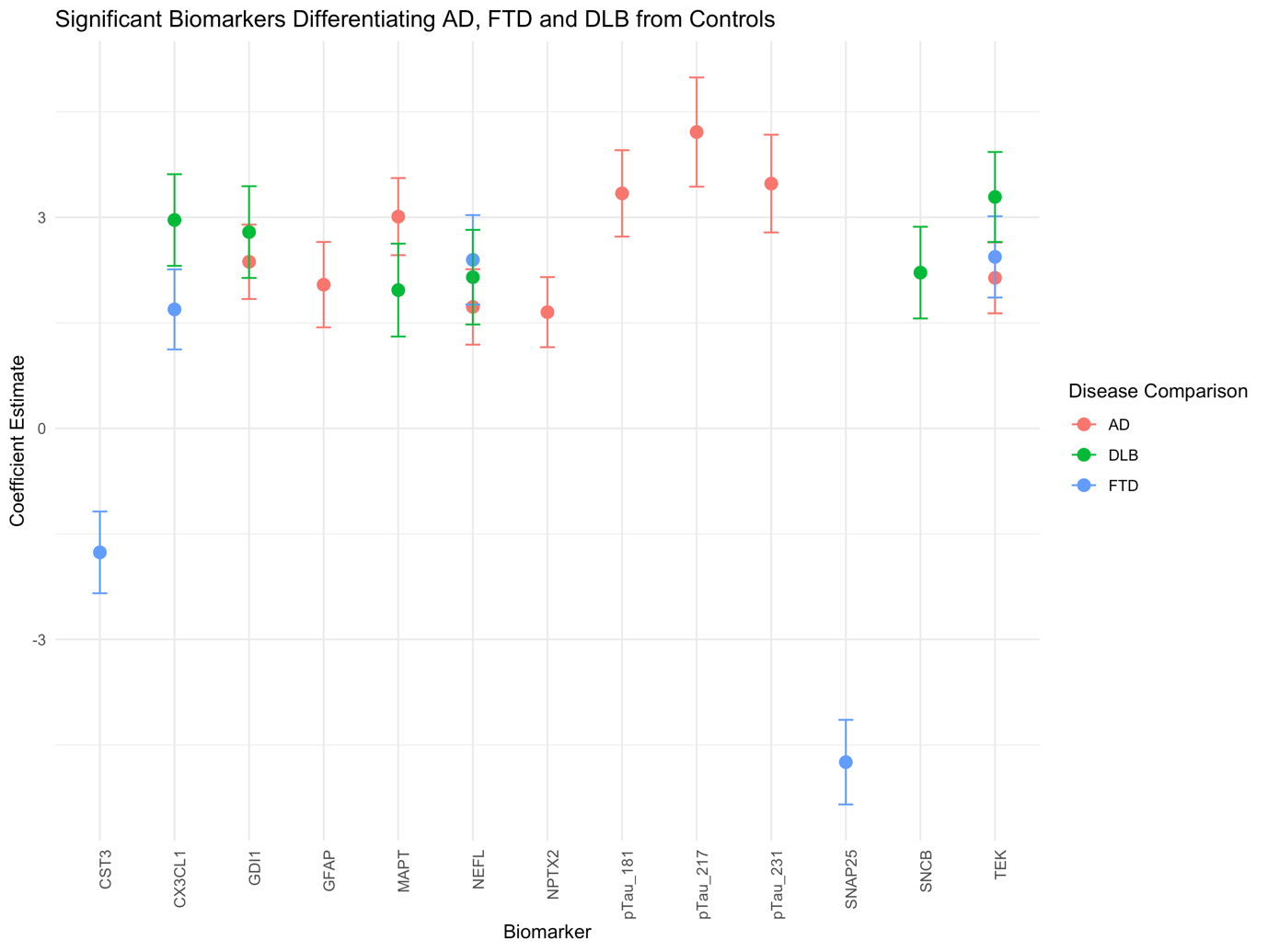


***Supp material 7.*** Multinomial Logistic Regression Coefficients for Biomarkers Differentiating AD, DLB and FTD from A-T-N- MCI. Biomarkers that above zero are increased in the disease group compared to A-T-N- MCI. Biomarkers that below zero are decreased in the disease group compared to A-T-N- MCI. Biomarkers that below zero are increased in AD compared to DLB or FTD. FTD: Frontotemporal Dementia, DLB: Dementia with Lewy Bodies, AD: Alzheimer’s Disease, MCI: Mild cognitive impairment, CST3: cystatin C, CX3CL1: chemokine (C-X3-C motif) ligand 1 (fractalkine), GDI1: GDP dissociation inhibitor 1, GFAP: glial fibrillary acidic protein, MAPT: microtubule-associated protein tau, NEFL: neurofilament light chain, NPTX2: neuronal pentraxin-2, pTau181: phosphorylated Tau-181, pTau217: phosphorylated Tau-217, pTau231: phosphorylated Tau-231, SNAP25: synaptosomal-associated protein 25, SNCB: beta synuclein, TEK: tyrosine kinase.

***
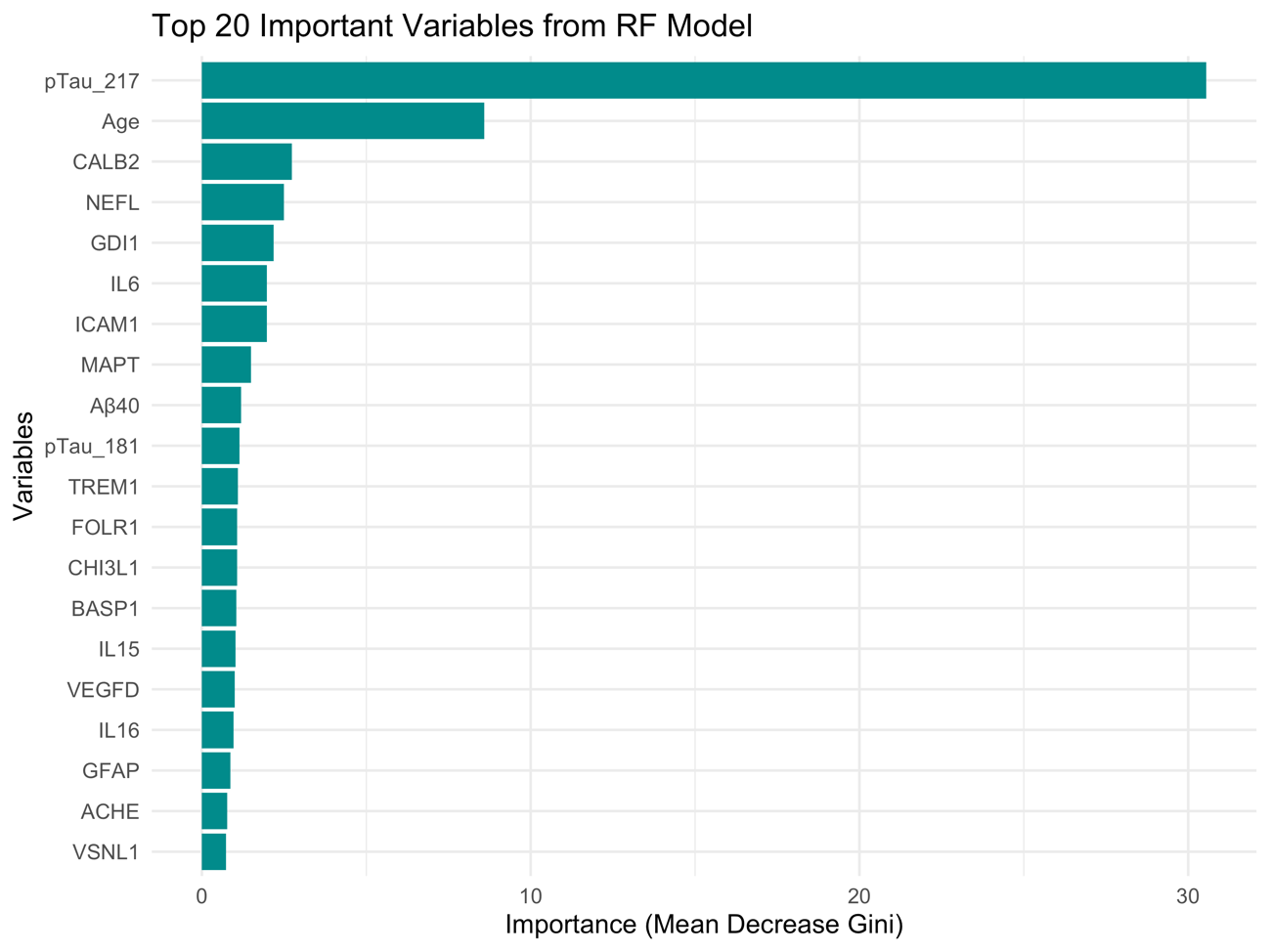
***

**Supp material *8.*** Top 20 Important Variables for Differentiating AD, DLB, and FTD using a Random Forest Model. The plot ranks the top 20 most important variables based on the Mean Decrease Gini score from the Random Forest (RF) model. pTau_217: phosphorylated tau-217, CALB2: calretinin, NEFL: neurofilament light chain, GDI1: GDP dissociation inhibitor 1, IL6: interleukin 6, ICAM1: intercellular adhesion molecule 1, MAPT: microtubule-associated protein tau, Aβ40: amyloid beta 40, pTau_181: phosphorylated tau-181, TREM1: triggering receptor expressed on myeloid cells 1, FOLR1: folate receptor 1, CHI3L1(YKL40): chitinase-3-like protein 1, BASP1: brain acid soluble protein 1, IL15: interleukin 15, VEGFD: vascular endothelial growth factor D, IL16: interleukin 16, GFAP: glial fibrillary acidic protein, ACHE: acetylcholinesterase, VSNL1: visinin like 1


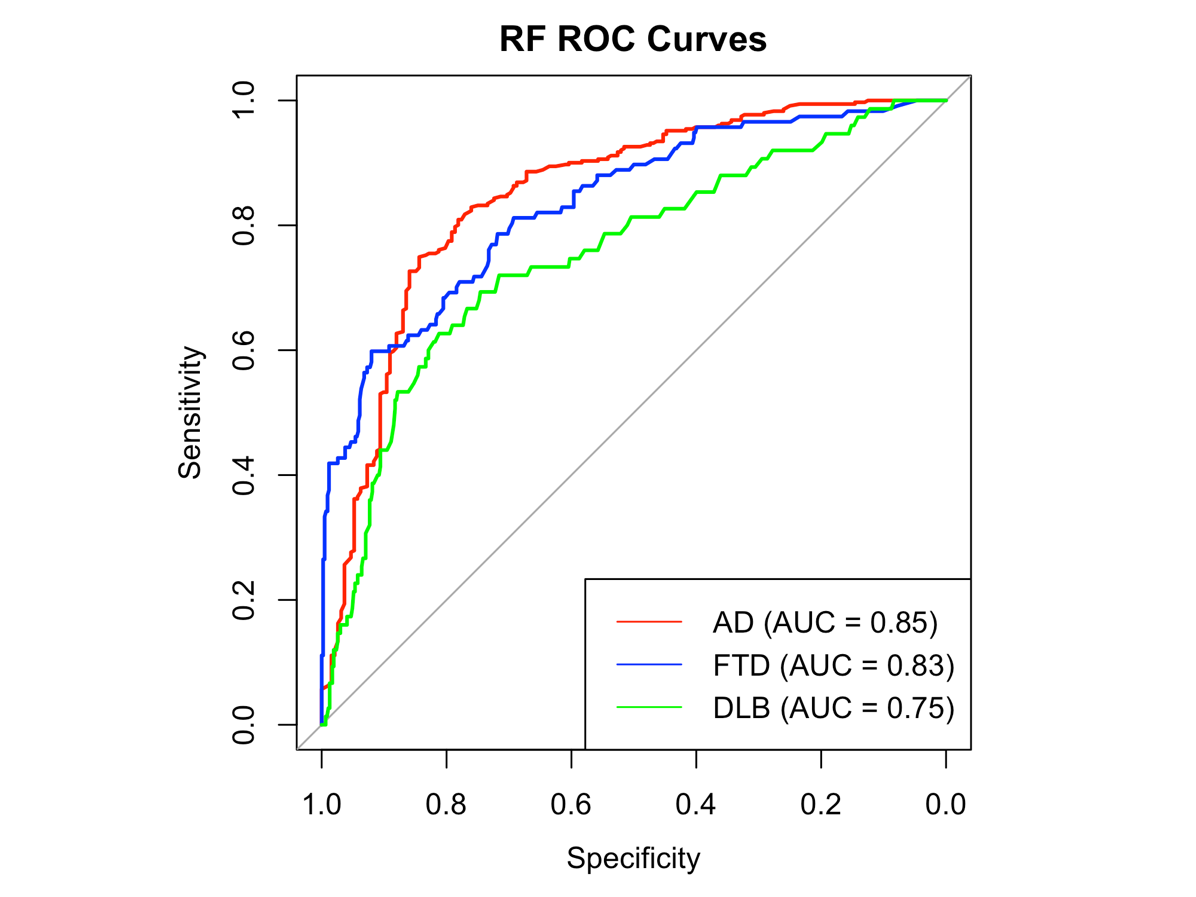


**Supp material *9.*** Random forest (RF) analysis ROC curve for dementia diagnosis*.* The ROC curves display the diagnostic performance of a model combining all plasma biomarkers, age, and sex for predicting AD, DLB, FTD diagnoses. The AUC for AD, FTD, and DLB are 0.85, 0.83 and 0.75, respectively. AD: Alzheimer's Disease, DLB: Dementia with Lewy Bodies, FTD: Frontotemporal Dementia, AUC: Area Under the Curve


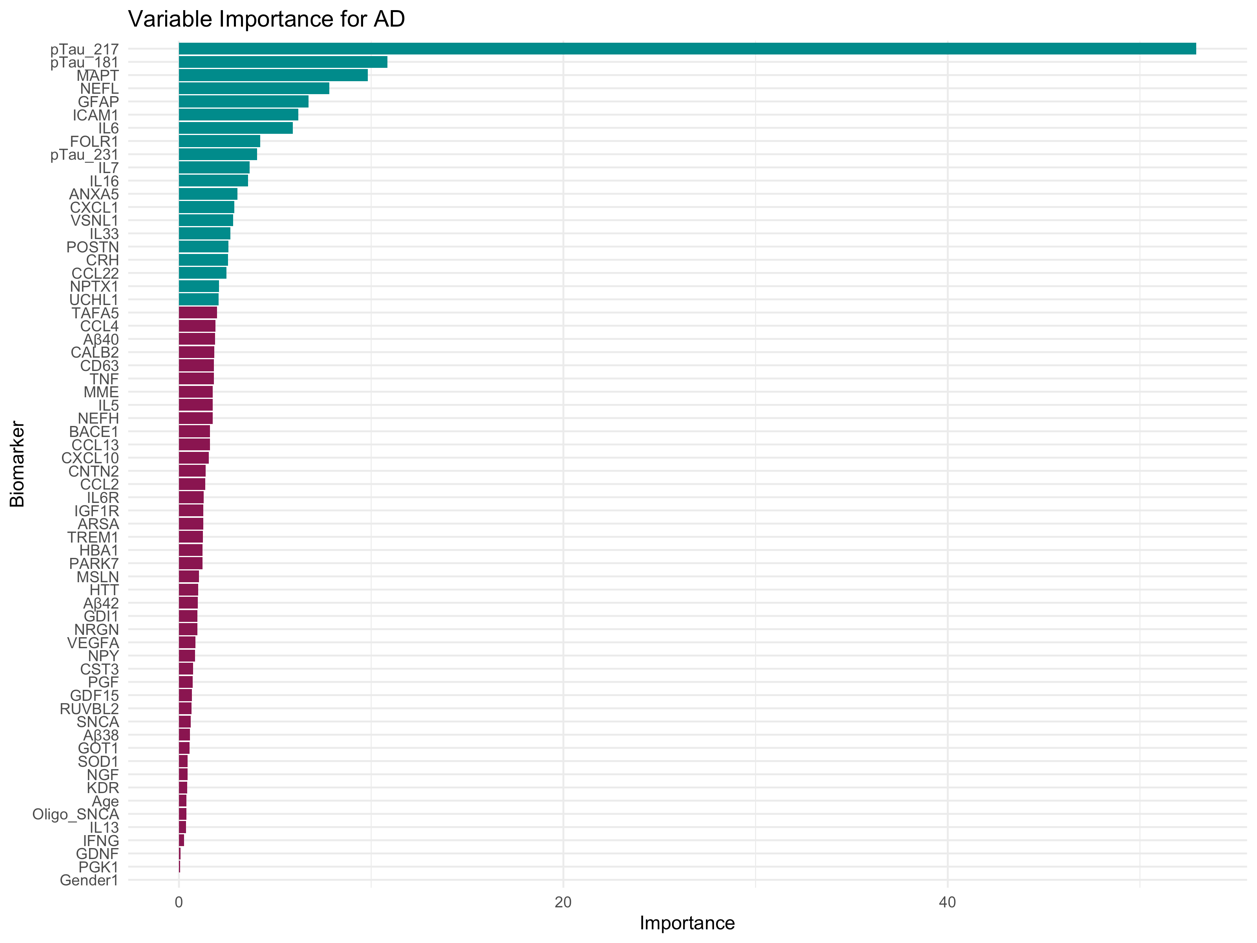


***Supp. Material 10***. Importance of biomarkers for AD diagnosis, based on random forest model. This figure ranks biomarkers by their importance in distinguishing Alzheimer’s Disease from other conditions in a random forest model. Biomarkers are ordered by their relative importance score, highlighting the key variables that contribute to the model's predictive accuracy. pTau_217: phosphorylated tau-217, pTau_181: phosphorylated tau-181, MAPT: microtubule-associated protein tau, NEFL: neurofilament light chain, GFAP: glial fibrillary acidic protein, ICAM1: intercellular adhesion molecule 1, IL6: interleukin 6, FOLR1: folate receptor 1, pTau_231: phosphorylated tau-231, IL7: interleukin 7, IL16: interleukin 16, ANXA5: annexin A5, CXCL1: CXC motif chemokine ligand 1, VSNL1: visinin-like protein 1, IL33: interleukin 33, POSTN: periostin, CRH: corticotropin releasing hormone, CCL22: C-C motif chemokine ligand 22, NPTX1: neuronal pentraxin-1, UCHL1: ubiquitin carboxy-terminal hydrolase L1

*
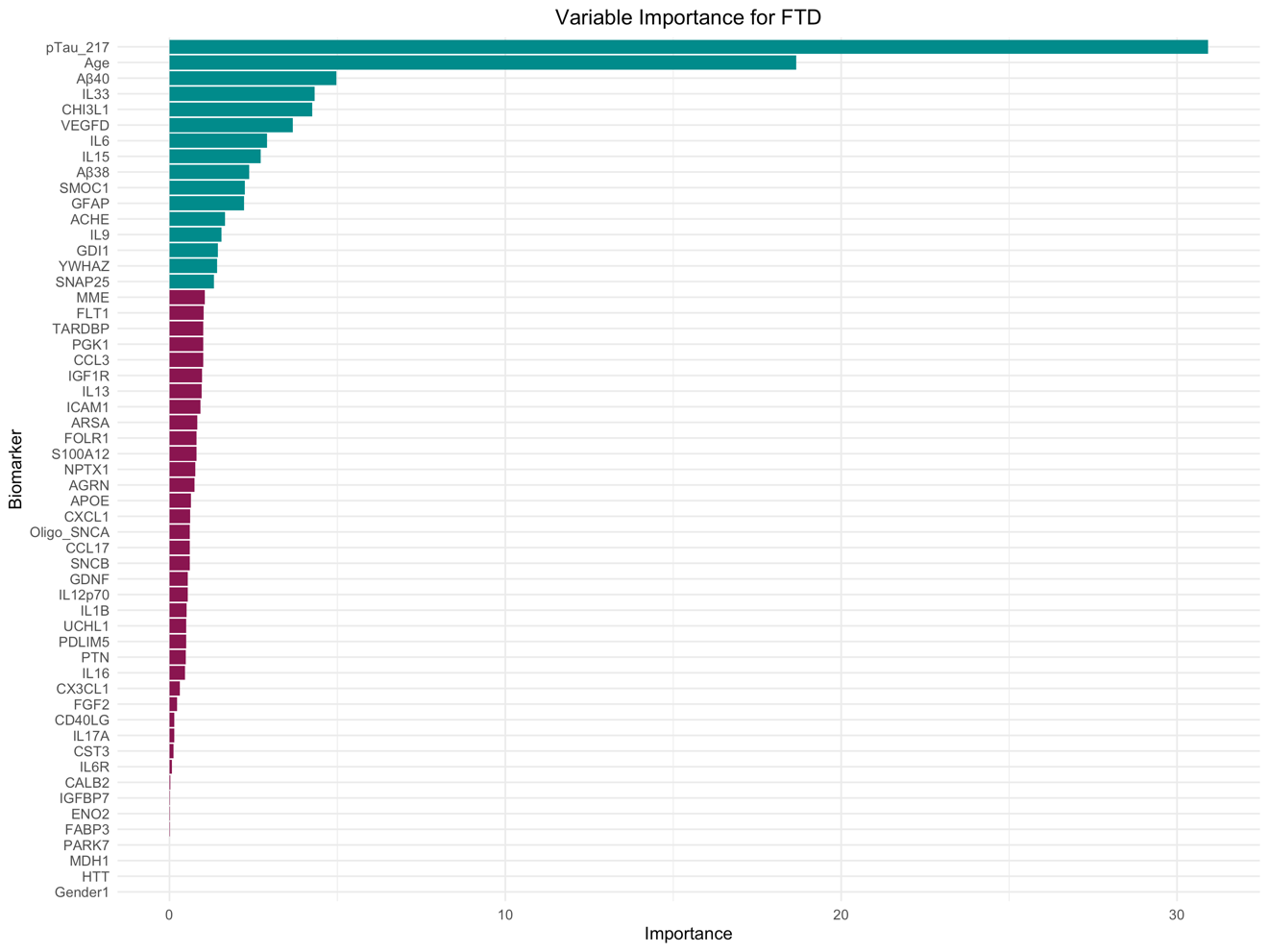
*

***Supp. Material 11****.* Importance of biomarkers for Frontotemporal Dementia (FTD) diagnosis, based on random forest model. This figure ranks biomarkers by their importance in distinguishing FTD from other conditions in a random forest model. Biomarkers are ordered by their relative importance score, highlighting the key variables that contribute to the model's predictive accuracy. pTau_217: phosphorylated tau-21, Aβ40: amyloid-beta 40, IL33: interleukin 33, CHI3L1: chitinase-3-like protein 1, VEGFD: vascular endothelial growth factor D, IL6: interleukin 6, IL15: interleukin 15, Aβ38: amyloid-beta 38, SMOC1: SPARC-related modular calcium-binding protein 1, GFAP: glial fibrillary acidic protein, ACHE: acetylcholinesterase, IL9: interleukin 9, GDI1: GDP dissociation inhibitor 1, YWHAZ: tyrosine 3-monooxygenase activation protein zeta, SNAP25: synaptosomal-associated protein 25.

***
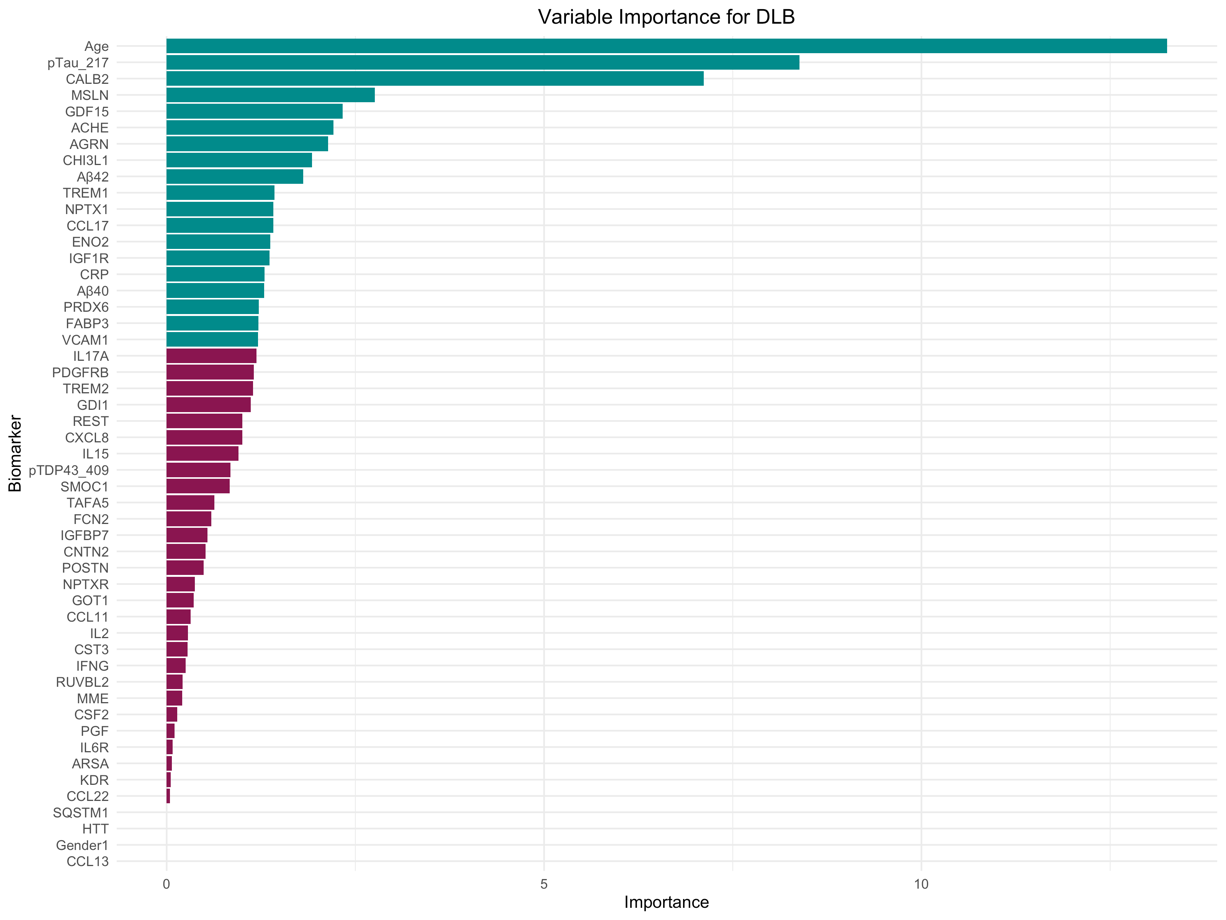
***

***Supp. Material 12***. Importance of biomarkers for Dementia with Lewy Bodies (DLB) diagnosis, based on random forest model. This figure ranks biomarkers by their importance in distinguishing DLB from other conditions in a random forest model. Biomarkers are ordered by their relative importance score, highlighting the key variables that contribute to the model's predictive accuracy. pTau_217: phosphorylated tau-217, CALB2: calbindin 2, MSLN: mesothelin, GDF15: growth differentiation factor 15, ACHE: acetylcholinesterase, AGRN: agrin, CHI3L1: chitinase-3-like protein 1, Aβ42: amyloid-beta 42, TREM1: triggering receptor expressed on myeloid cells 1, NPTX1: neuronal pentraxin 1, CXCL17: C-X-C motif chemokine ligand 17, ENO2: enolase 2, IGF1R: insulin-like growth factor 1 receptor, CRP: C-reactive protein, Aβ40: amyloid-beta 40, PRDX6: peroxiredoxin 6, FABP3: fatty acid-binding protein 3, VCAM1: vascular cell adhesion molecule 1


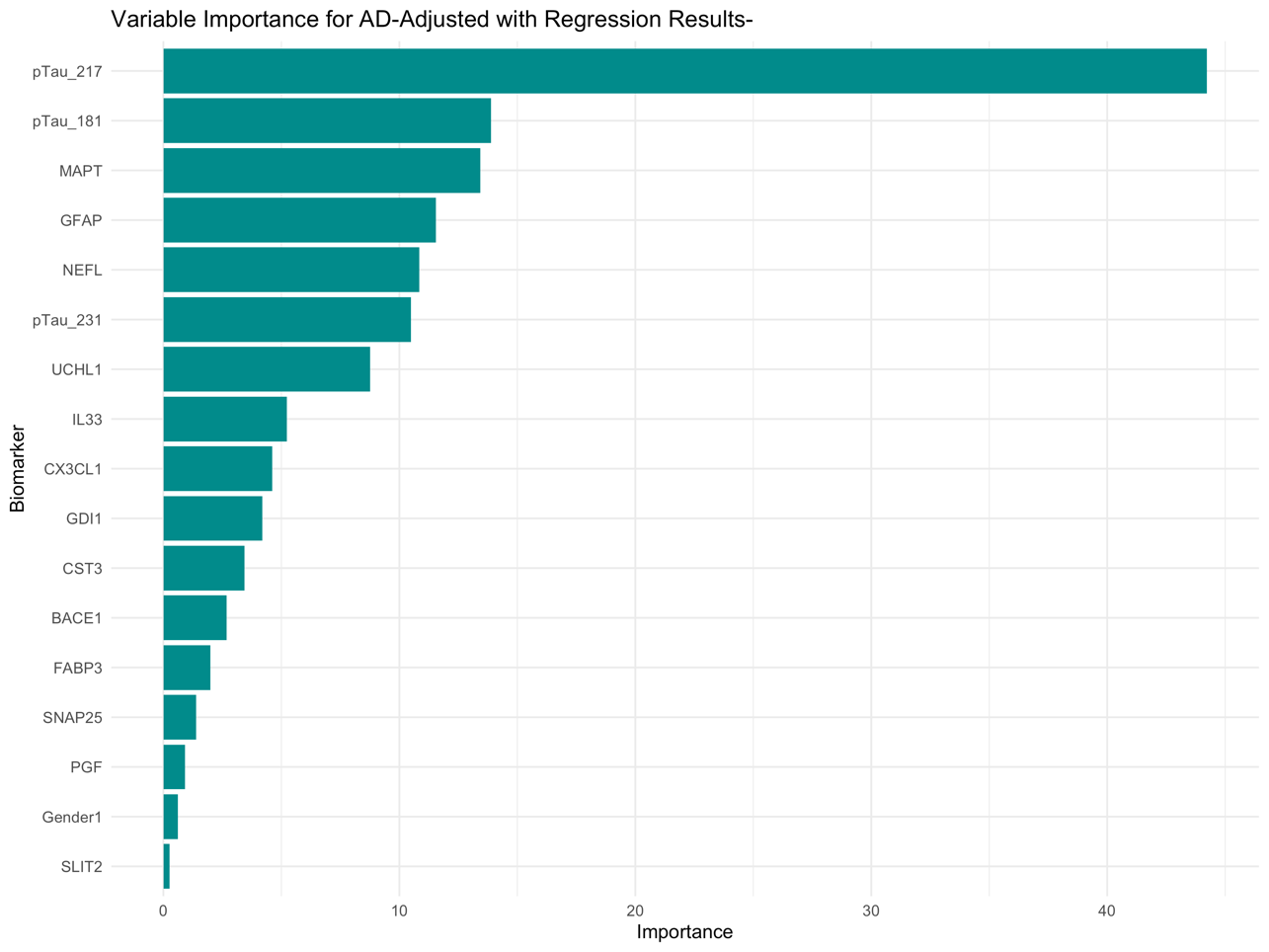


***Supp. Material 13****.* This figure shows the variable importance for distinguishing Alzheimer Dementia (AD), as determined by a Random Forest model, adjusted with regression results. The bars represent the importance of the top biomarkers in classifying AD patients, based on their Mean Decrease in Gini index. pTau_217: phosphorylated tau-217, pTau_181: phosphorylated tau-181, MAPT: microtubule-associated protein tau, GFAP: glial fibrillary acidic protein, NEFL: neurofilament light chain, pTau_231: phosphorylated tau-231, UCHL1: ubiquitin carboxyl-terminal hydrolase L1, IL33: interleukin-33, CXCL1: CXC motif chemokine ligand 1, GDI1: GDP dissociation inhibitor 1, CST3: cystatin C, BACE1: beta-site amyloid precursor protein cleaving enzyme 1, FABP3: fatty acid-binding protein 3, SNAP25: synaptosomal-associated protein 25, PGF: placental growth factor, SLIT2: slit guidance ligand 2.


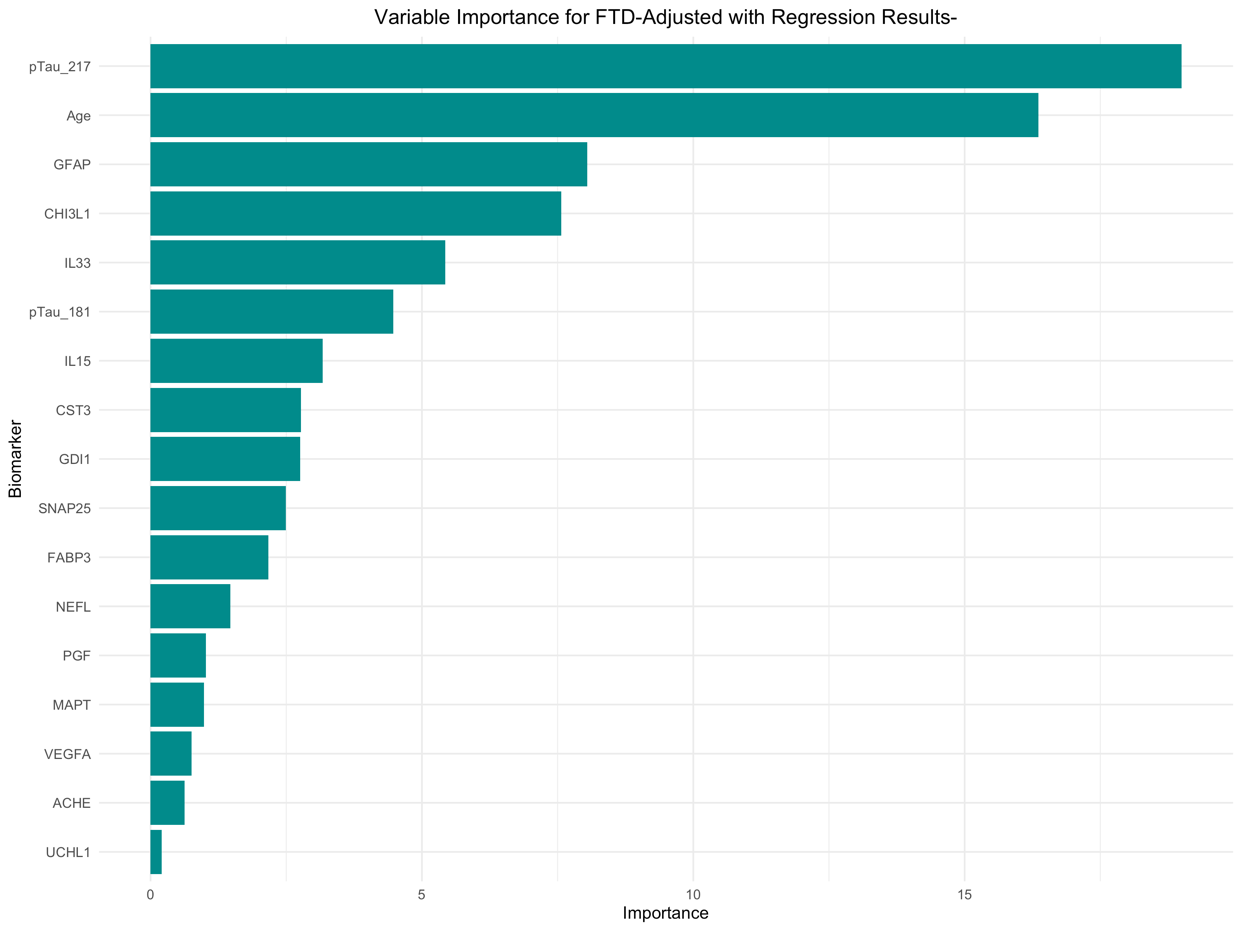


***Supp. Material 14****.* This figure shows the variable importance for distinguishing Frontotemporal Dementia (FTD), as determined by a Random Forest model, adjusted with regression results. The bars represent the importance of the top biomarkers in classifying FTD patients, based on their Mean Decrease in Gini index. pTau_217: phosphorylated tau-217, GFAP: glial fibrillary acidic protein, CHI3L1: chitinase-3-like protein 1, IL33: interleukin-33, pTau_181:phosphorylated tau-181, IL15: interleukin-15, CST3: cystatin C, GDI1: GDP dissociation inhibitor 1, SNAP25: synaptosomal-associated protein 25, FABP3: fatty acid-binding protein 3, NEFL: neurofilament light chain, PGF: placental growth factor, MAPT: microtubule-associated protein tau, VEGFA: vascular endothelial growth factor A, ACHE: acetylcholinesterase, UCHL1: ubiquitin carboxyl-terminal hydrolase L1.

*
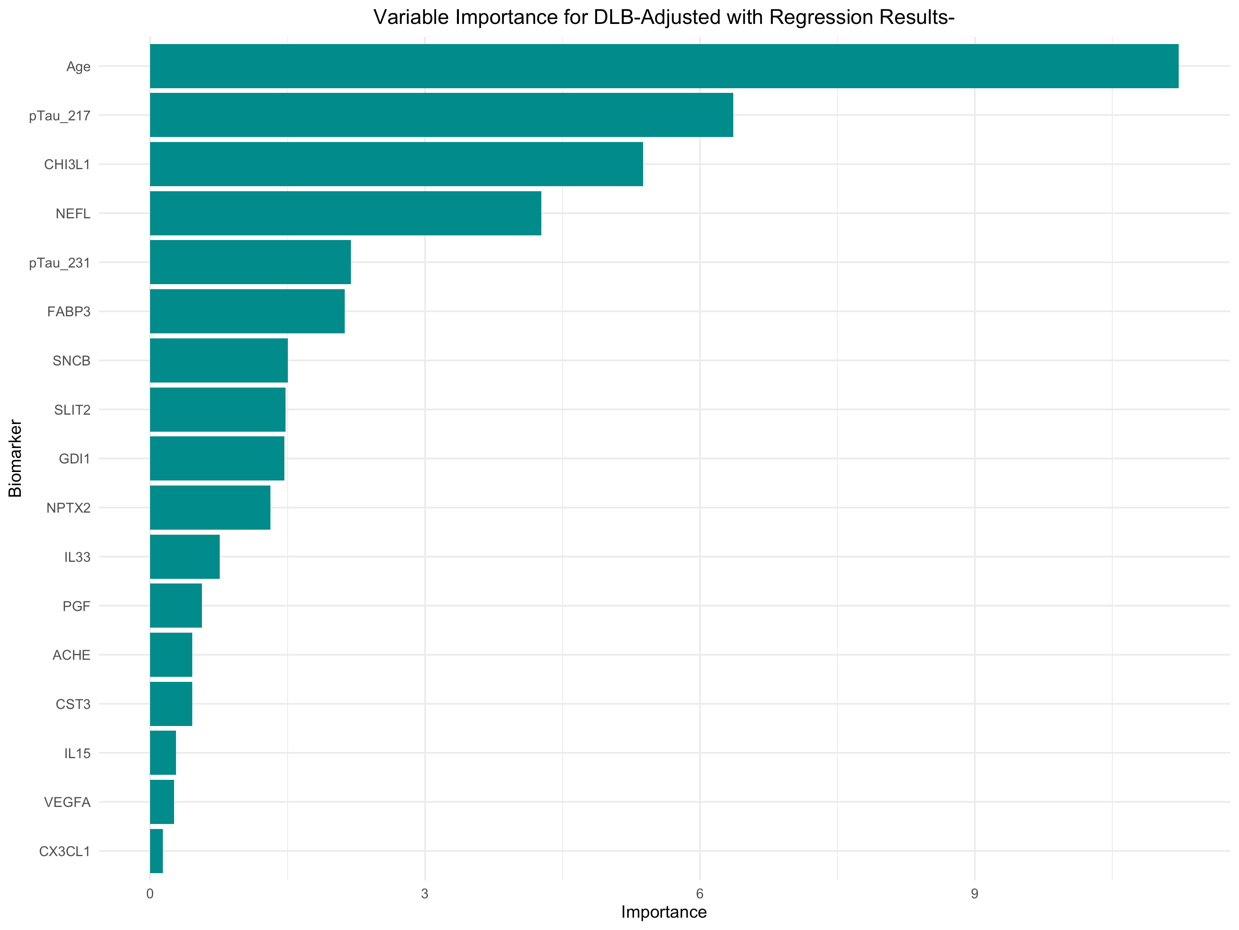
*

***Supp. Material 15****.* This figure shows the variable importance for distinguishing Dementia with Lewy Bodies (DLB), as determined by a Random Forest model, adjusted with regression results. The bars represent the importance of the top biomarkers in classifying DLB patients, based on their Mean Decrease in Gini index. Tau_217: phosphorylated tau-217, CHI3L1: chitinase-3-like protein 1, NEFL: neurofilament light chain, pTau_231: phosphorylated tau 231, FABP3: fatty acid-binding protein 3, SNCB: beta-synuclein, SLIT2: slit guidance ligand 2, GDI1: GDP dissociation inhibitor 1, NPTX2: neuronal pentraxin 2, IL33: interleukin-33, PGF: placental growth factor, ACHE: acetylcholinesterase, CST3: cystatin C, IL15: interleukin-15, VEGFA: vascular endothelial growth factor A, CX3CL1: fractalkine.

***
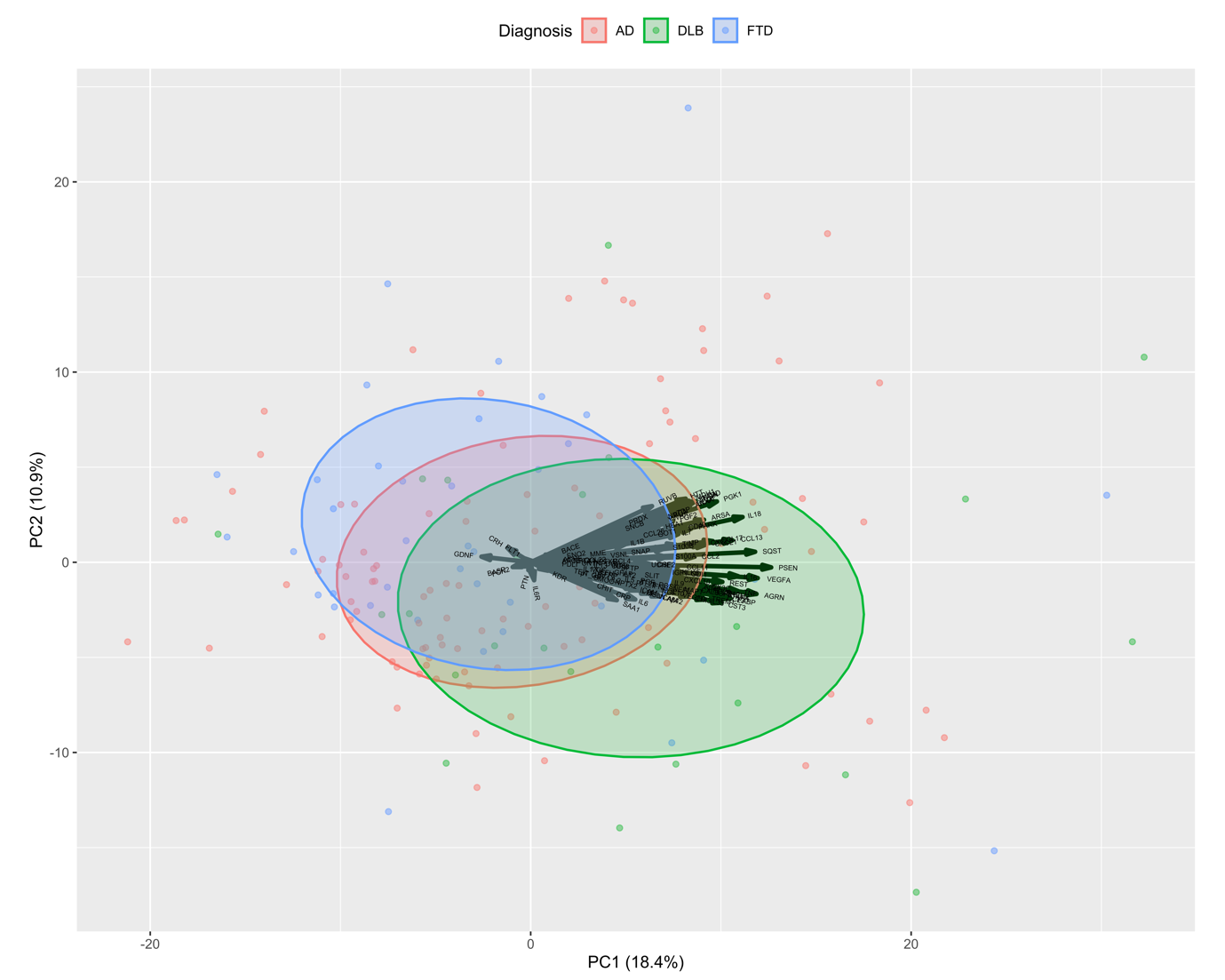
Supp. Material 16.*** Principal Component Analysis (PCA) Biplot of Plasma Biomarker Data. This PCA biplot visualizes the distribution of plasma biomarker profiles across different dementia subtypes, including Alzheimer's disease (AD, red), Dementia with Lewy bodies (DLB, green), and Frontotemporal dementia (FTD, blue). The x-axis represents the first principal component (PC1), explaining 18.4% of the variance, while the y-axis represents the second principal component (PC2), accounting for 10.9% of the variance. Each point corresponds to an individual sample, coloured according to diagnosis. The black arrows indicate the loadings of individual biomarkers, with their direction and length reflecting their contribution to the principal components. While some degree of separation between diagnostic groups is observed, overlap remains, suggesting shared biomarker profiles among the dementia subtypes.

***
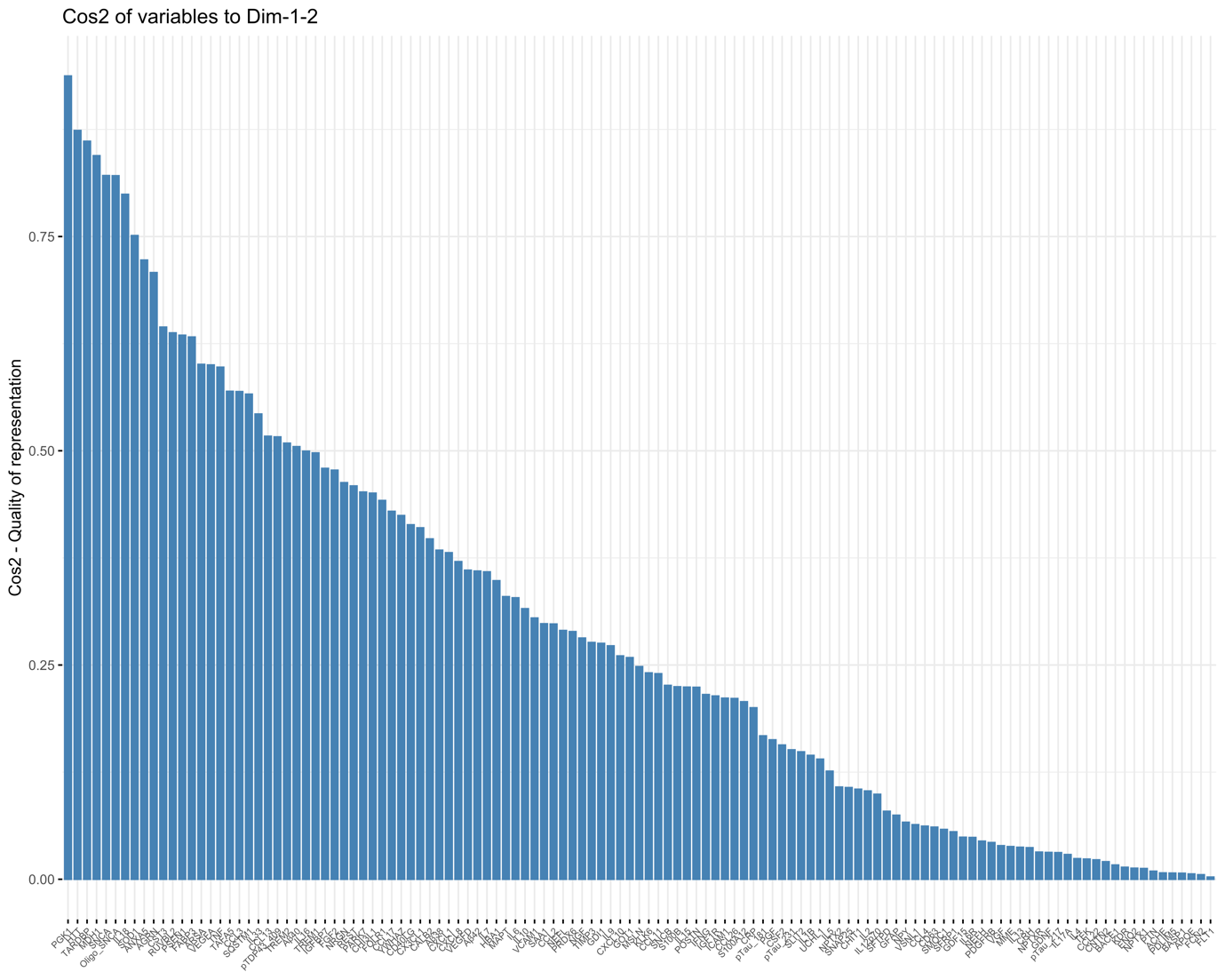
Supp. Material 17.*** The bar plot of the loadings responsible for PC1 and PC2. This figure represents the cos² values (quality of representation) of variables on the first two principal components (PC1 and PC2) from a principal component analysis (PCA). The x-axis lists the biomarkers, while the y-axis represents the cos² values, which indicate how well each variable is represented in the first two principal components. Higher cos² values suggest that a biomarker is well-explained by PC1 and PC2, contributing significantly to the variance in the data. Conversely, lower cos² values indicate that a biomarker is less important in defining these principal components.

***
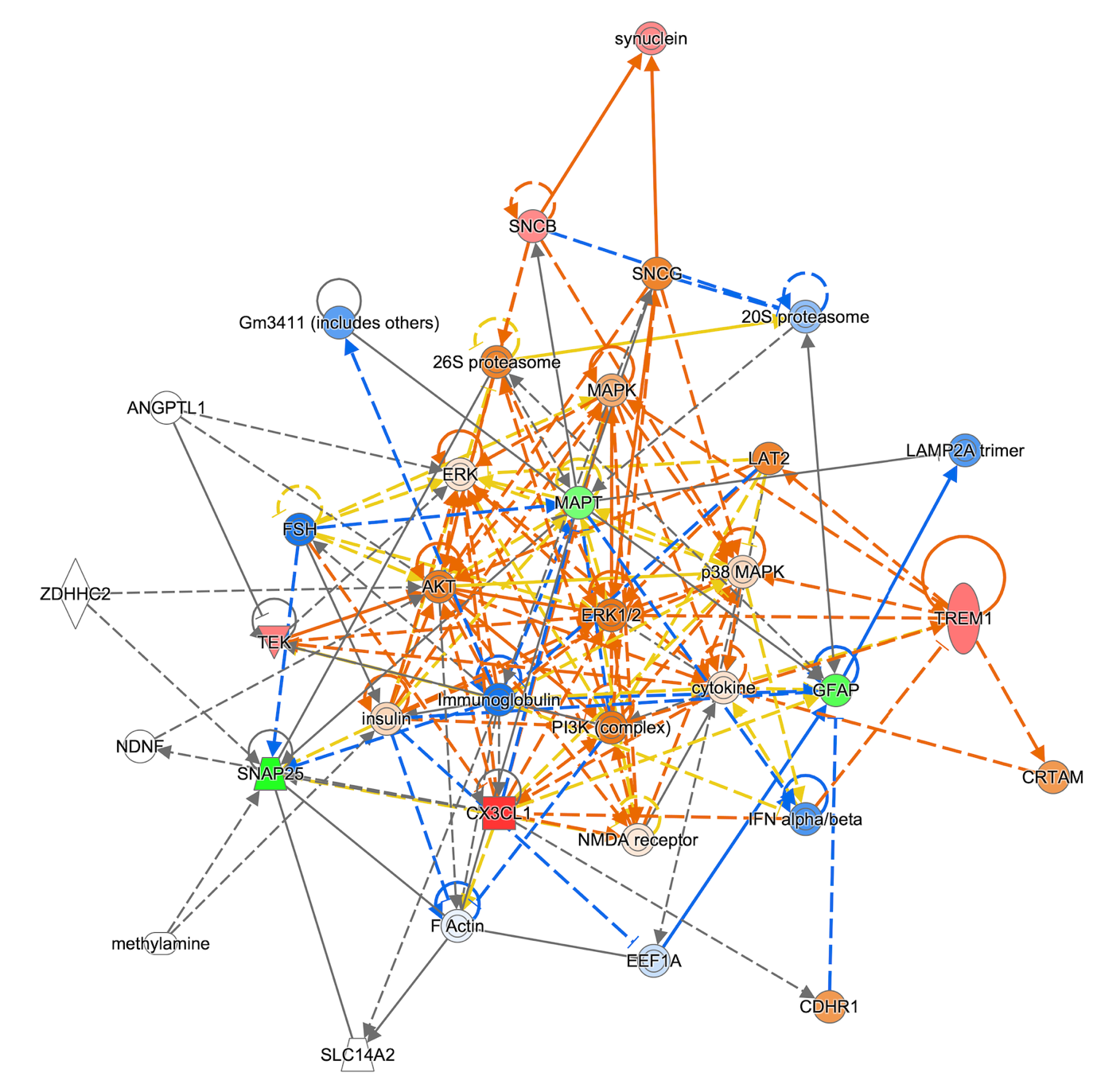
***

***Supp. Material 18.*** Ingenuity Pathway Analysis (IPA) analysis of differently expressed biomarkers between AD and DLB. The dense connectivity of the DLB network underscores the complexity of synaptic and inflammatory pathways such as chaperone mediated autophagy through lysosome-associated membrane protein type 2A (LAMP2A). The strong presence of TREM1 and cytokine signaling pathways reflects the inflammatory milieu characteristic of DLB. Notable upstream regulators include AKT1 and ERK1/2, which integrate synaptic and survival signals. The direction of the change (inhibition) also indicates these pathways are more prominently activated in AD, the relative activation of these pathways could indicate a compensatory mechanism in response to the accumulation of amyloid-β and hyperphosphorylated tau.

***
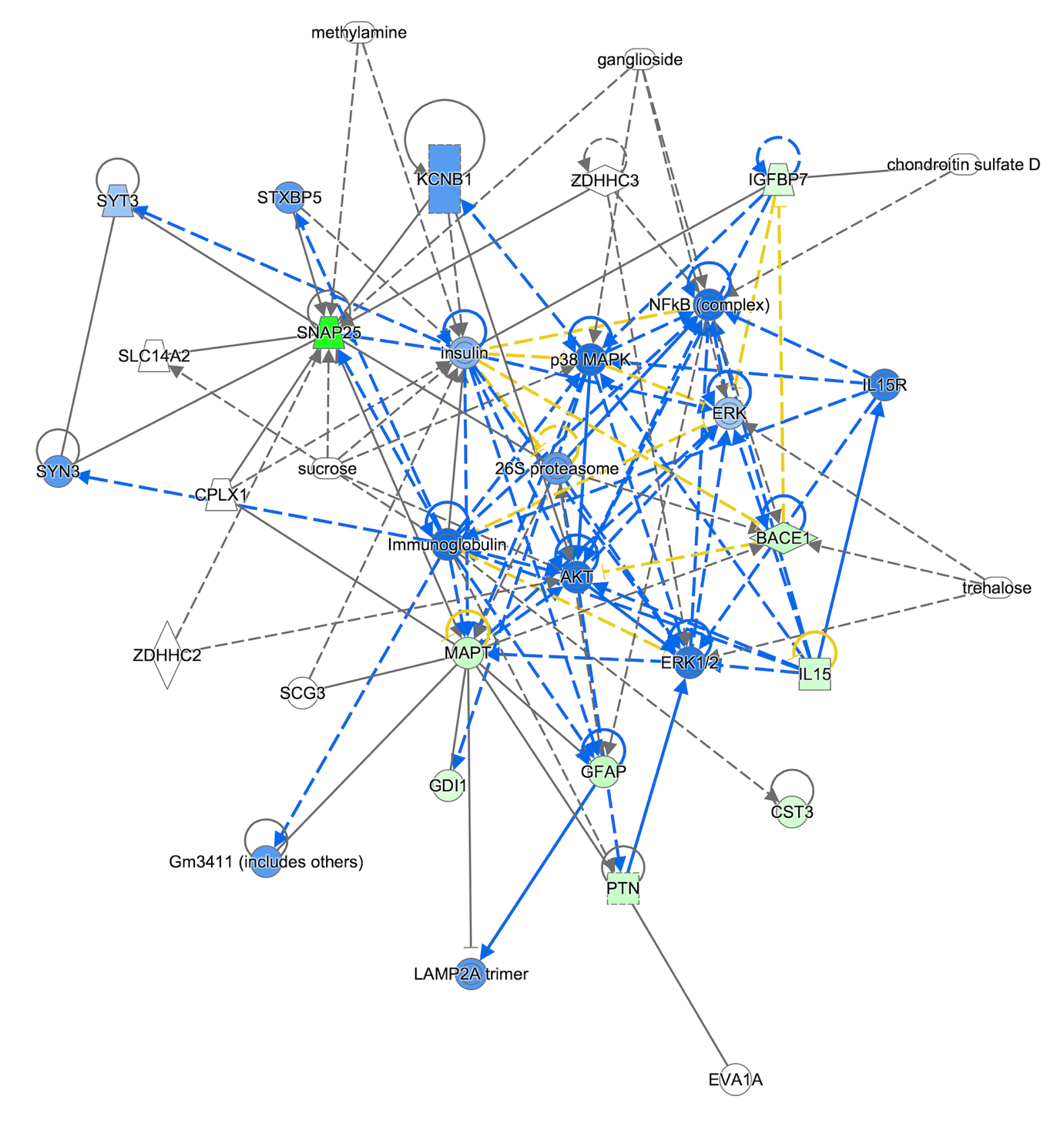
Supp. Material 19.*** Ingenuity Pathway Analysis (IPA) analysis of differently expressed biomarkers between AD and FTD. The FTD network is more focused, with fewer upstream regulators and pathways compared to DLB. MAPT is the dominant regulator, reflecting the centrality of tau pathology in FTD. Synaptic signaling is less pronounced, consistent with FTD's pathophysiology. The FTD network displayed fewer inflammatory pathways but more focused interactions involving ERK 1/2 signaling.


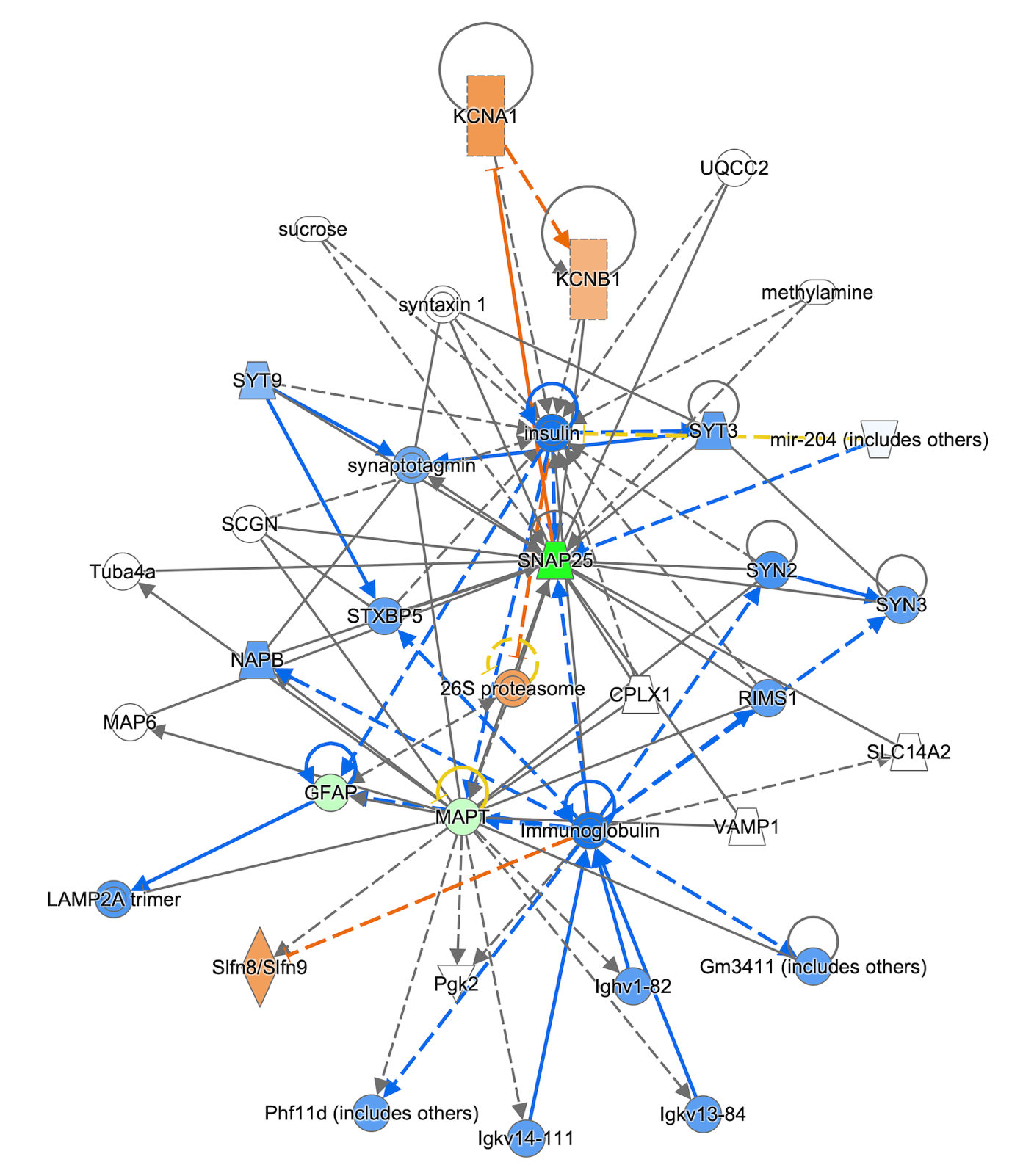


***Supp. Material 20.*** Ingenuity Pathway Analysis (IPA) analysis of differently expressed biomarkers between AD, DLB and FTD. The combined network reveals shared mechanisms, including SNAP25, MAPT, and GFAP, but retains condition-specific features. This highlights both the overlap and divergence in disease mechanisms, with synuclein and PI3K/AKT pathways specific to DLB and tau-related pathways predominating in FTD.
